# Light harvesting FIT DNA hybridization probes for brightness-enhanced RNA detection†

**DOI:** 10.1039/d4sc06729k

**Published:** 2024-12-02

**Authors:** Amal Homer, Andrea Knoll, Uschi Gruber, Oliver Seitz

**Affiliations:** a Institut für Chemie, Humboldt-Universität zu Berlin 12489 Berlin Germany oliver.seitz@hu-berlin.de

## Abstract

Fluorogenic hybridization probes are essential tools in modern molecular biology techniques. They allow detection of specific nucleic acid molecules without the need to separate target-bound from unbound probes. To enable detection of targets at low concentration, fluorogenic probes should have high brightness. Here, we report the development of RNA hybridization probes (RNA FIT probes) that use smart quenching and a light harvesting principle to enhance the brightness of fluorescence signaling. The signaling mechanism is based on FRET between brightly emitting donor dyes and a fluorescent base surrogate, such as quinoline blue (QB) or thiazole orange (TO). In the single-stranded state, QB/TO nucleotides fluoresce weakly and quench the fluorescence of the donor dyes. Upon target recognition, QB/TO stack with adjacent base pairs, resulting in enhanced fluorescence quantum yields. The donor dyes are blue-shifted by only 5–20 nm relative to the QB/TO nucleotides, allowing simultaneous excitation of both dye groups with efficient energy transfer. The combined photon absorption results in exceptionally bright FIT probes. This feature facilitated the detection of RNA target in undiluted cell lysates. The present study examines the utilization of probes to detect mRNA targets in live T cells using flow cytometry.

## Abstract 2

Light harvesting FIT probes contain a fluorescent base surrogate acting as a smart quencher of bright auxiliary dyes. After hybridization, simultaneous excitation of both dye groups increases the brightness of hybridization-induced fluorescence.

## Graphical abstract

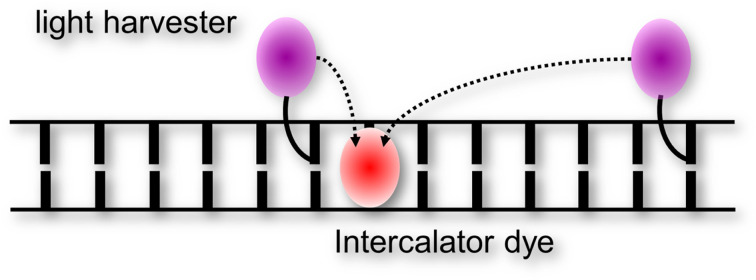

## 1 Introduction

Fluorogenic oligonucleotide hybridization probes enable the detection of DNA/RNA when unbound probes cannot be separated from bound probes, for example, in order to detect specific nucleic acid sequences during polymerase chain reaction (PCR) assays or in living cells.^1–3^ The performance of a fluorogenic probe is defined by two key parameters: the degree of fluorescence increase upon target binding and the brightness of the fluorescence signal. The latter is important when probe/target concentrations are low in relation to autofluorescence of the matrix, a challenge that arises for measurements of cell lysates, cells or even tissues.

One of the most frequently utilized detection principles is based on the distance-dependent interaction between a fluorescence donor and a fluorescence acceptor. Molecular Beacon-type probes are designed to assume a hairpin shape that permits energy transfer from one fluorophore to the other chromophore.^4,5^ Binding of the target induces a conformational rearrangement which increases the distance between the two chromophores and thereby minimizes energy transfer. A disadvantage of detection principles relying on large scale conformational changes is that even interactions with nucleic acid-binding proteins can induce donor emission.^6^ In the majority of cases, read-out is focused on donor emission. In the bound state, the acceptor chromophore plays a relatively minor role; it can be considered a mere cargo that, at worst, limits the maximum attainable intensity of the donor signal.

**Fig. 1:**
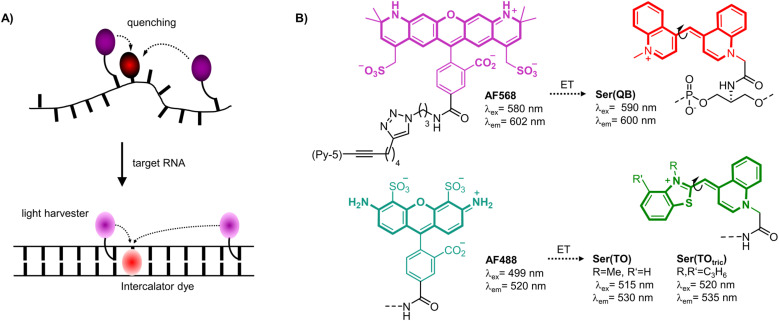
(A) Principle of light-harvesting FIT probes. An intercalator dye (red) is linked as fluorescent base surrogate and quenches the emission of the light harvester dye (magenta) in the single-stranded date. After hybridization with target RNA the light harvester transfers excitation energy to the fluorescent base surrogate, which fluoresces due to restriction of twisting motions around the central methine bridge. (B) The absorption maxima of the light harvester dyes such as AF568 and AF488 are blue-shifted by 5–20 nm relative to FIT dyes such as Ser(QB) or Ser(TO) enabling efficient energy transfer (ET).

Herein we introduce a fluorogenic probe technology in which energy transfer between two or more dyes persists in both the unbound and bound states. This approach enables all dyes to contribute to emission of the target-bound probe and thereby enhance brightness of signaling. The method is based on FIT probe methodology, which was introduced by us to increase the sequence specificity of nucleic acid detection and facilitate RNA live cell imaging. FIT probes are oligonucleotide probes with an intercalator dye of the thiazole orange (TO) family inserted as a surrogate nucleobase that serves as a hybridization reporter.^7–11^ Upon target recognition, the TO-like intercalator dye is embedded within the double helical base stack (Fig. 1A). The resulting restriction of torsional motions around the central methine bridge prolongs the lifetime of the excited state, and hence increases fluorescence. The FIT probe methodology was used previously for realtime PCR,^12,13^ live cell RNA imaging,^14–21^ RNA double strand detection,^22^ fluorogenic aptamers^23,24^ and was also considered for detecting RNA editing.^25–28^

The brightness of fluorescent signaling by FIT probes is limited by the TO-like dye incorporated as a fluorescent base surrogate. TO and quinoline blue (QB) emit in a useful spectral range (TO: 530 nm, QB: 600 nm) and, when incorporated into DNA FIT probes, provide up to 16-fold (TO) or 195-fold (QB) increases of fluorescence upon hybridization. However, brightness is limited by moderate quantum yields (TO: *ϕ* ≤ 0.5; QB: *ϕ* ≤ 0.6). To improve signal intensity, we introduced two TO or QB dyes.^27^ Although sequence specificity was high, brightness enhancements were rather small due to self-quenching. Herein we present a new approach which involves coupling of light-collecting “auxiliary dyes” to create extremely bright FIT probes. The intercalator dyes QB,^18,29^ TO^7,10^ and a tricyclic benzothiazole containing TO derivative (TO_tric_)^30^ were designed to serve two distinct functions. In addition to their established role as hybridization reporters, the fluorescent base surrogates were hypothesized to function as smart acceptors of Förster resonance energy transfer (FRET) from bright Alexa Fluor (AF) dyes (568 or 488, Fig. 1B right).^31^ These auxiliary dyes were selected to enable simultaneous excitation of both the auxiliary dye and the hybridization reporter while permitting partial overlap (Fig. S6†) with the absorption of the fluorescence base surrogate. The donor excitation maxima were blue-shifted by 5–20 nm relative to the hybridization reporter. As result, excitation energy can be transferred required that the two dyes are positioned in sufficient proximity. However, emission can only occur if the torsions of the TO-like hybridization reporter dye around the methine bridge are restricted by formation of the probe-target complex. In addition, in the single stranded state, the probe can adopt conformations that allow direct contact of the two dyes. Therefore, in the absence of target, the hybridization reporter acts as quencher. In presence of target, intercalation prevents direct contact and the auxiliary dyes serve as light collectors that increase the extinction coefficient of the base surrogates acting as hybridization reporter. The base surrogates can be regarded as “smart” FRET acceptors, given that they act as quenchers in the absence of target and as emitters when target is present.

## 2 Results and discussion

**Fig. 2:**
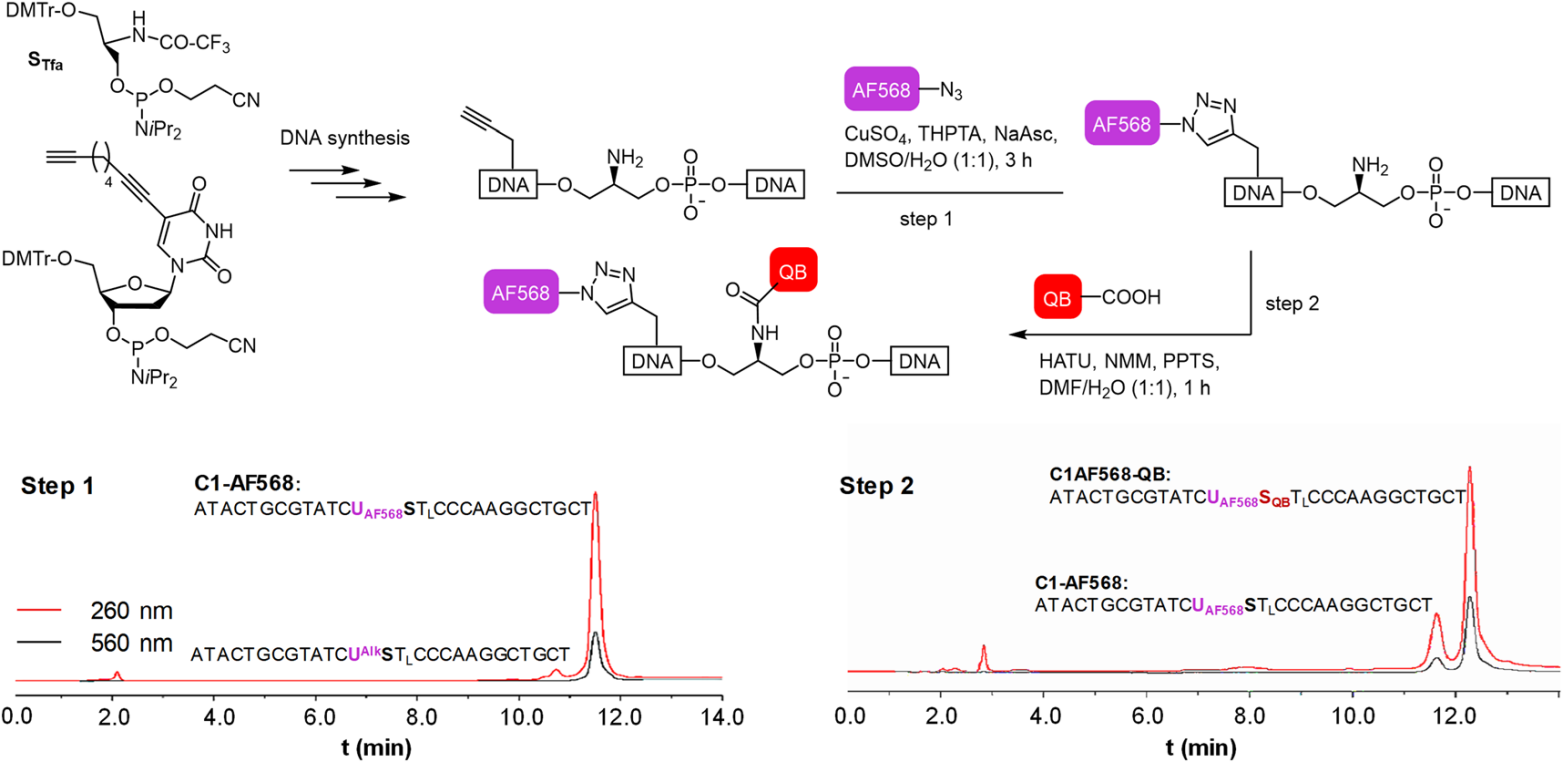
Step wise labelling of oligonucleotides with AF568 and QB *via* Cu-click (step 1) and amide bond formation (step 2) to form the light harvesting FIT probes. HPLC traces show the analysis of aliquots from the reaction mixture after step 1 (left) and step 2 (right).

We commenced our study by preparing DNA FIT probes (Fig. 2) targeting the CDR3 regions of T cell receptor (TCR) mRNA in the CCRF-CEM T cell line. The serinol-linked QB nucleotide S_QB_ (see Fig. 1B) was introduced at a central position of oligonucleotide probes. S_QB_ was flanked by an LNA nucleotide, which increases QB brightness most probably due to local rigidification of the probe-target duplex.^15^ During DNA synthesis, alkyne-modified uridine was coupled at varied distances to the N-Tfa-protected serinol nucleotide (S_Tfa_). Ammonia cleavage provided oligonucleotides offering alkynyl groups and the serinol amino group for post-synthetic introduction of dyes. First, Cu-click (step 1) was used to conjugate the auxiliary dyes. For a combination with the QB dye, we selected the sulfonated AF 568 dye and assumed that the net negative charge would minimize interactions with negatively charged DNA/RNA. The conjugation reaction proceeded smoothly in 94% yield for C1-AF568. The HPLC-purified material was used in the coupling of the QB dye (step 2). Employing HATU as activator of the carboxyl function and PPTS as enhancer of QB solubility the coupling succeeded with 73% yield for C1-AF568-QB.^30^ This synthesis protocol proved robust and provided rapid access to multi-labelled oligonucleotides.

**Fig. 3:**
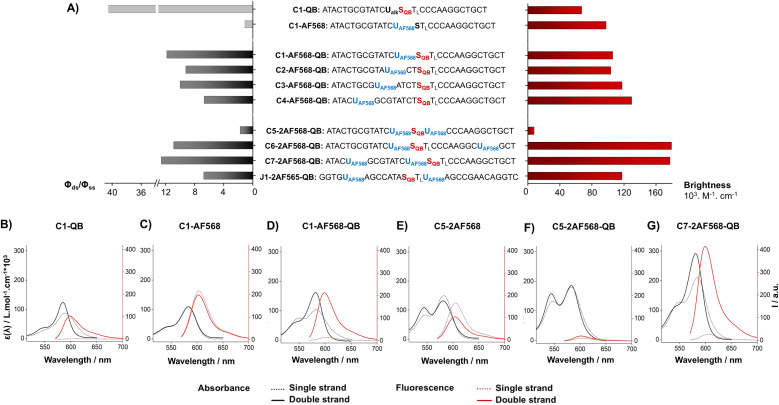
(A) Comparison of hybridization-induced enhancement in fluorescence quantum yield (*ϕ*_ds_/*ϕ*_ss_, black and grey bars left) and maximum achievable brightness (red bars, right) in double-stranded state for FIT probes containing only QB, only AF568 or both dyes. (B–G) Comparison of absorbance spectra (black) and fluorescence spectra (red) of indicated probes before (dotted lines) and after (solid lines) hybridization with the complementary RNA. Subscript L mark LNA nucleotides, conditions: 200 nM probe and 4 eq of RNA target in PBS (100 mM NaCl, 10 mM Na_2_HPO_4_, pH 7) at 25 °C. *λ*_ex_ = 560 nm, *λ*_em_ = 570−800 nm.

**Fig. 4:**
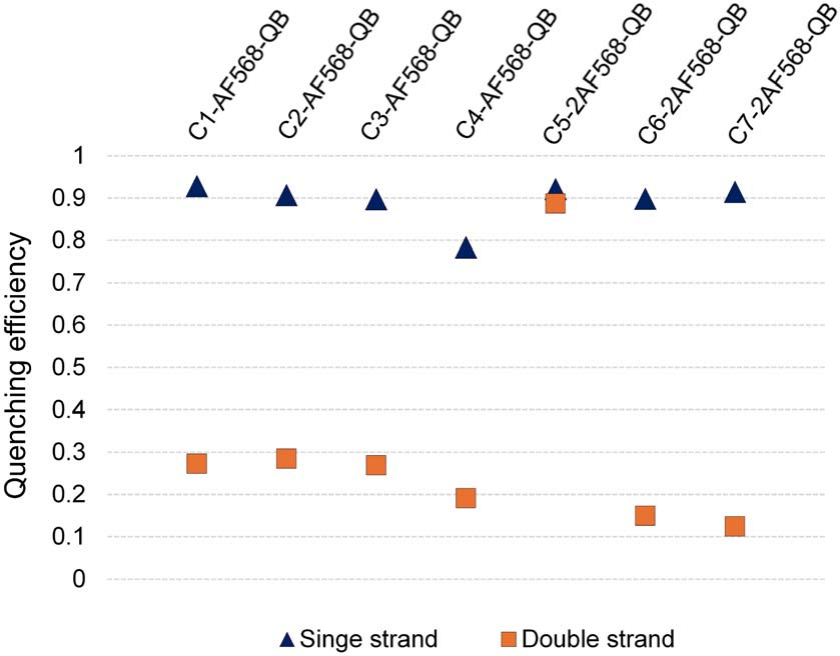
Quenching efficiencies (*Q*) for the light harvesting FIT probes before (blue triangles) and after (orange squares) hybridization with target RNA. *Q* = 1 – *F*_(AF-QB)_/(*F*_(AF)_ + *F*_(QB)_), where *F*_(AF-QB)_ is the integrated fluorescence intensity of the light harvesting FIT probes containing AF568 and QB, *F*_(QB)_ and *F*_(AF)_ are the integrated fluorescence intensities of the corresponding QB-FIT probes and AF-568 containing probes upon excitation at 560 nm.

Hybridization of the QB-containing FIT probe C1-QB with the RNA target was accompanied by a 44-fold increase in fluorescence quantum yield (Fig. 3A). For comparison, we prepared probe C1-AF568, which tethers an AF568 dye to the 5-position of a uridine. This probe has near unity quantum yield (Table S3†) and is much brighter than C1-QB, but non-responsive (Fig. 3B, C and Tables S2, S3†), indicating efficient dye solvation in both single and double stranded states. Of note, introduction of the QB dye in C1-AF568-QB rendered emission from AF568 responsive to hybridization, as shown by the low fluorescence of the single stranded probe (Fig. 3D). The absorption band at 550 nm indicates dye–dye contact in the absence of target RNA. Importantly, target-bound dual labelled probe C1-AF568-QB is brighter than the mono-labelled probe C1-QB (Fig. 3A, right). Of note, quantum yields of both single stranded and target-bound states (Table S3†) remained at the level of the C1-QB probe indicating that excitation energy is efficiently transferred from AF568 to QB. Excitation spectra would provide little additional information due to the extreme similarity of the donor and acceptor spectra. We conclude that bright emission of target- bound C1-AF568-QB results from a combination of extinction coefficients of the dye constituents. The overlap between the emission spectrum of AF568 and the absorption spectrum of QB (Fig. S6†) should result in sufficient Förster radii (see Table S9† for an estimation). This should make it possible to position AF568 on nucleotides that are not in close proximity to the QB dye and still maintain energy transfer. In oligonucleotides C2-, C3- and C4-AF568-QB, the AF568 dye was separated from the QB hybridization reporter by 2, 4 or 8 spacer nucleotides (Fig. 3A). The added extinction coefficients of the two dyes ensured an enhanced brightness of the probes while maintaining low single-strand fluorescence due to QB-induced quenching of AF568 emission in absence of target (Fig. 3A and Tables S2, S3†). A comparison of the four probes shows that fluorescence turn-on is increased with decreasing AF568-QB distance (Fig. 3A, left). The accompanying decrease in single strand fluorescence (Table S2†) suggests that proximity facilitates quenching of AF568 by QB. It is instructive to plot the overall quenching efficiency for single stranded and target-bound forms of the AF568-QB probes (Fig. 4). This plot puts their emission intensity in relation to the emission intensity that would be expected if fluorescence emission of single labelled AF568 and QB probes were additive. In the single stranded state, arranging the dyes in 1, 3 or 5 nucleotides distance quenching efficiency is very high with values >93%. This shows that non-intercalated QB is an efficient quencher of AF568 emission. For C4-AF568-QB the quenching efficiency drops to 80%, which is plausible given the increased distance (9 nt) between the two dyes. Target-bound probes show much lower quenching due to a 45-fold increase of QB quantum yield upon hybridization. The fact that quenching efficiency does not reach zero values suggests that energy transfer occurs also in the target-bound state. It is not possible to selectively excite and read the emission of the donor dye or the acceptor dye. Therefore, conventional methods for determining FRET efficiencies are not applicable. For an estimation, we compare the quantum yield of donor–acceptor-labelled probes with that of the corresponding probes containing only a single dye. At 100% FRET efficiency, the quantum yield should be governed by the acceptor dye, whereas an arithmetic mean of the quantum yields would be expected if the two dyes emit independently. This analysis suggests a high efficiency of FRET in C1-AF568-QB (*ϕ*_ds_ = 0.56 *vs. ϕ*_ds_ = 0.93 and 0.53 for C1-AF568 and C1-QB, respectively), which decreases substantially for C4-AF568-QB (*ϕ*_ds_ = 0.65 *vs. ϕ*_ds_ = 0.95 and 0.53 for C4-AF568 and C4-QB, respectively). We conclude, that the two dyes begin to emit independently as the FRET efficiency is affected by the increasing distance. As a result, C4-AF568-QB is brighter than C1-AF568-QB.

Next, we examined probes containing two auxiliary dyes in addition to the QB nucleotide. Due to the highest fluorescence turn-on, the first auxiliary dye was placed adjacent to QB. In C5-2AF568-QB, the two AF568 dyes were placed as 3′ and 5′ next neighbours of the QB base surrogate. This probe showed characteristics of aggregation-induced quenching (Fig. 3F). Quantum yields were low (*ϕ*_ss_ = 0.02, *ϕ*_ds_ = 0.04), and quenching efficiency (Fig. 4) estimated from fluorescence of single labelled probes was high in both single and double stranded states. Also in absence of the QB dye in C5-2AF568, fluorescence quantum yields were much lower than observed for single AF568-labelled probes (*ϕ*_ds_(C5-2AF568) = 0.26 *vs. ϕ*_ds_(C1-AF568) = 0.92). This and the absorption spectra (peak at 540 nm, Fig. 3E) suggest that proximity allows the AF568 dyes to readily form an H-dimer.^32^ In stark contrast, probes C6-2AF568-QB and C7-2AF568-QB (Fig. 3A) showed exceptional brightness of up to 179 000 M^−1^ cm^−1^ (Table S4†). These probes contained the second auxiliary dye in 10 or 7 nt distance to the first AF568 dye. Importantly, in the single stranded state, the single QB dye was still able to quench emission of the two AF568 dyes, enabling 14- to 17-fold enhancements in fluorescence upon hybridization (Fig. 3G and S2†). In J1-2AF568-QB, which is specific for the TCR CDR3 region expressed by Jurkat cells, we again placed the first AF568 near the QB dye, and the second AF568 in a larger distance to prevent aggregation-induced self quenching. In this sequence context too, introduction of a QB base surrogate and two light harvesting AF568 dyes provided the desired features of high brightness in the target-bound state while fluorescing poorly in the absence of target (Fig. S2†).

With proof of principle established, we moved on to the development of probes that would allow measurements in the presence of nucleolytic enzymes in cells and cell lysates (Table 1). 2′-OMe modifications were introduced into the backbone. In most cases, the resulting RNA FIT probes provided even higher brightness and higher extents of hybridization-induced fluorescence enhancements than the DNA FIT probes (Table 1). To explore the generality of the light harvesting approach we also prepared RNA FIT probes based on thiazole orange nucleotides, which emit in the green spectral region. TO and its recently discovered tricyclic analogue TO_tric_ were combined with AF488 dyes.^30^ The excitation maxima of AF488 are blue-shifted by 15–20 nm relative to TO and TO_tric_. Again, with brightness values up to 99 000 M^−1^ cm^−1^ the presence of the auxiliary dyes ensured high emission intensity of target-bound probes while TO and TO_tric_ retained the ability to quench the emission in the single stranded state.

**Table 1:** 2′-OMe gapmer FIT-probes in the red (QB)^a^ and green (TO, TOtric)^b^ fluorescence channels targeted against RNA

Probe	*ϕ*_ss_	*ϕ*_ds_	*ϕ*_ds_/*ϕ*_ss_	Br^c^/10^3^
C1-AF568-QB-OMe	0.036	0.52	14.4	109.6
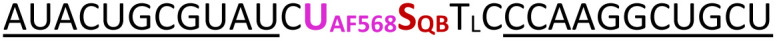				
C7-2AF568-QB-OMe	0.049	0.65	13.3	224.8
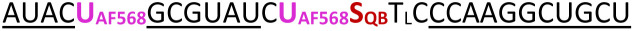				
JK1-2AF568-QB-OMe	0.048	0.56	11.7	155.2
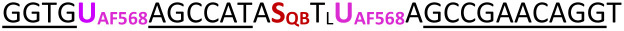				
C1-AF488-TO_tric_-OMe	0.041	0.49	11.8	60.0
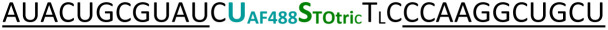				
C7-2AF488- TO_tric_ -OMe	0.050	0.46	9.18	96.5
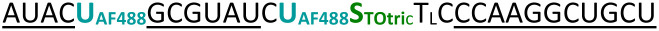				
JK1-2AF488-TO_tric_ OMe	0.045	0.44	9.6	99.4
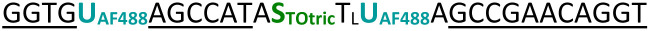				

a *λ*_ex_ = 560 nm, *λ*_em_ = 570–800 nm.

b *λ*_ex_ = 485 nm, *λ*_em_ = 500–700 nm.

c At *ε*_max_ in M^−1^ cm^−1^. Underlines mark 2′-OMe modifications.

**Fig. 5:**
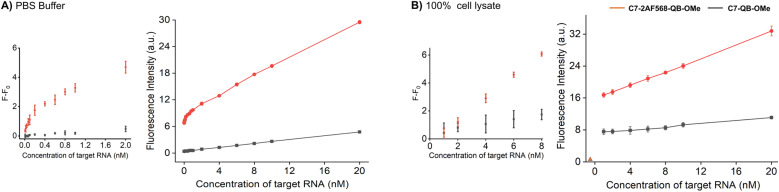
RNA detection in (A) PBS buffer and (B) 100% cell lysate (obtained from CCRF-CEM cells (ACC 240, Leibniz Institute DSMZ-German Collection of Microorganisms and Cell Cultures)). Fluorescence intensities measured for the C7-QB-OMe (black line) and C7-2AF568-QB-OMe at different concentrations of synthetic target RNA. Fluorescence intensity for the cell lysate without probe is denoted by the orange triangle in (B). *λ*_ex_ = 560 nm, *λ*_em_ = 600 nm.

Increased brightness of fluorescence signals is advantageous when the concentration of the probe is low and/or when background is high due to matrix autofluorescence. In this regard, it is instructive to compare probes C7-2AF568-QB-OMe and C7-QB-OMe, which have different characteristics. While C7-QB-OMe provides the highest enhancement of fluorescence intensity upon hybridization (75-fold for C7-QB-OMe *vs.* 18-fold for C7-2AF568-QB-OMe), C7-2AF568-QB-OMe is much brighter (Br_max_ (C7-2AF568-QB-OMe) = 224.800 M^−1^ cm^−1^*vs.* Br_max_ (C7-QB-OMe) = 72.500 M^−1^ cm^−1^). This proved important for the detection of small amounts of the target RNA. Increasing amounts of target RNA were added to probes C7-2AF568-QB-OMe and C7-QB-OMe in PBS and the fluorescence intensity at 600 nm was measured (Fig. 5A). Light-harvesting probe C7-2AF568-QB-OMe afforded a 40 pM limit of detection (LOD) compared to 2 nM with the traditional FIT probe C7-QB-OMe. The increased sensitivity of the light-harvesting probe is mainly due to a smaller influence of deviations in the background-corrected fluorescence *F* − *F*_0_. For example, at 1 nM target the standard deviation amounts to 17% of the signal provided by C7-QB-OMe but only to 3% for C7-2AF568-QB-OMe.

Next, we evaluated the probes in undiluted cell lysates, which is a challenging matrix due to its autofluorescence and viscosity. Plotting the target-dependent fluorescence increase against target concentration showed again a larger slope for the light-harvesting FIT probe (Fig. 5B), which indicates a higher sensitivity for RNA detection also in this matrix. The two auxiliary dyes in C7-2AF568-QB-OMe provided a 4-fold improvement in the limit of detection (LOD: C7-2AF568-QB-OMe, 2 nM; C7-QB-OMe, 8 nM). The lower performance of probes in lysate has been observed previously.^27^ Single stranded FIT probes tend to show stronger emission in viscous environment and standard deviations are higher, which might be due to a viscosity response.^33^

**Fig. 6:**
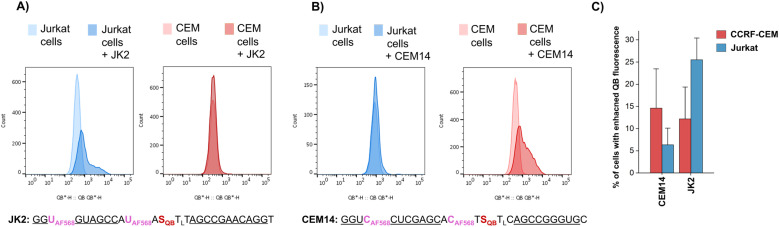
Flow cytometric evaluation of light-harvesting FIT probes in the detection of TCR mRNA in Jurkat cells and CCRF CEM cells. Histograms of Jurkat cells or CCRF CEM cells after mock eletroporation and after electroporating with the (A) JK2 probe and the (B) CEM14 probe. (C) Comparison of the percentage of cells having fluorescence higher than autofluorescence after microporation (data from 5–6 replicates). Cell lines were originally purchased from the Leibniz Institute DSMZ-German Collection of Microorganisms and Cell Cultures: Jurkat (ACC 282) and CCRF-CM (ACC 240).

To further increase matrix complexity and explore scope and limitations, we examined FIT probes in the flow cytometry-based detection of mRNA in living cells. This application presents a significant challenge, particularly due to the low mRNA target concentrations and the difficulties in delivering probes into cells. This can lead to situations where either an inefficient number of probes have entered the cell to generate contrast against the background of cellular autofluorescence or an excess of probes remain unbound or mislocalize/aggregate when cell uptake is forced. We tested how FIT probes responded to different mRNA targets that encode the hypervariable CDR3 region of the T cell receptor (TCR). Microporation was used to deliver the probes into “difficult-to- transfect” T cells. Most of the cells (60–80% of 10^6^ cells, Fig. S7†) survived a 25 ms, single 240 V pulse, which allowed relatively weak staining. Jurkat cells were subjected to microporation with probes that were designed to target their TCR mRNA. In initial experiments, microporation was conducted using “traditional” FIT probes, which contained only QB dyes. However, no discernible difference was observed between cells treated with the probe and those subjected to mock microporation (data not shown), indicating that the fluorescence intensity was insufficient to yield meaningful results given the low efficiency of delivery. With the brighter light-harvesting probe JK2 it was possible to observe a population of Jurkat cells that exhibited fluorescence levels exceeding those observed in mock microporation (Fig. 6A). Flow cytometric analysis of six replicates demonstrated that following the delivery of JK2, 26% of cells exhibited higher fluorescence than the background (Fig. 6C). The mean fluorescence intensity of Jurkat cells was increased by an average of 177% (Table S10†). For comparison, delivery of JK2 into CCRF CEM cells (which express a different TCR) resulted in a lower increase of fluorescence intensity (+68%) and only 12% of the 6 × 10^5^ to 15 × 10^5^ live CCRF CEM cells analyzed exhibited higher than background fluorescence (see Fig. 6A, right for an example and Fig. 6C for averaged data). A control experiment used probe CEM14 (Fig. 6B) to target the TCR mRNA expressed by CCRF CEM cells. Microporation of CEM14 enhanced the mean fluorescence intensity of live CCRF CEM cells by 120%, compared to 67% of live Jurkat cells (Table S10†). On average, 15% of the CCRF CEM cells had higher fluorescence than mock-microporated cells, whereas only 6% of the CEM14-treated Jurkat cells exceeded background fluorescence (Fig. 6C).

It should be noted that the fold changes of mean fluorescence intensity induced by the probes were relatively small, probably due to the mild microporation protocol. Under these conditions, only a small fraction of the cells will receive probes. Harsher microporation would result in more efficient delivery. However, this increases the number of dead cells, which tend to acquire large amounts of probe and therefore give false positives. Further work is needed to improve delivery. Notwithstanding the challenges, the observation that (i) JK2 designed to target an mRNA target present in Jurkat cells but not in CCRF CEM cells induces higher fluorescence in Jurkat cells than in CCRF CEM cells and that (ii) CEM14 targeted against mRNA expressed by CCRF CEM cells but not by Jurkat cells increases stronger fluorescence increases in CCRF CEM cells suggests that the probes engage on the target.

## 3 Conclusions

We have developed a method to improve the brightness of fluorogenic hybridization probes (FIT probes) by introducing auxiliary dyes that serve as light harvesters. A distinctive feature of the probes is that the light-collecting dyes transfer excitation energy to a base surrogate, which only fluoresces if it can enter into stacking interactions with neighboring base pairs by forming the probe-target duplex. In the single-stranded state, dyes in the thiazole orange family act as fluorescence quenchers. Our data show that a QB or TO nucleotide can quench the fluorescence of two light-harvesting AlexaFluor dyes. It is conceivable that fluorescence quenching would be maintained if further AF dyes were introduced. The intercalation of the QB or TO dye, forcibly formed by hybridization with the target, closes quenching channels. The achievable quantum yields are limited by the QB or TO dyes, indicating energy transfer from the AF to the QB/TO dyes. Bright fluorescence obtained upon target binding is the result of more effective photon absorption. A prerequisite for this is to avoid quenching interactions between the light-harvesting dyes. Our data demonstrate that this is possible when the dyes are attached at a distance of 7–11 nucleotides to 5-alkynylpyrimidines by click reaction.

Due to the combination of bright fluorescence on the target and weak emission of the single strands, the light-harvesting FIT probes enabled the sensitive detection of RNA in challenging matrices such as pure cell lysate. In a scope and limitations study, we examined how the probes respond to the presence of target mRNA in live cells. In spite of challenges with the amount of probes delivered into T cells, the evidence suggests that the probes engage with their target. Firstly, probes designed to target an mRNA target present in Jurkat cells, but not in CCRF CEM cells, resulted in higher fluorescence levels in Jurkat cells than in CCRF CEM cells. Secondly, microporation of probes targeting the TCR mRNA of CCRF CEM cells induce higher fluorescence levels in CCRF CEM cells than in Jurkat cells. We conclude, the markedly enhanced brightness resulting from the incorporation of light-harvesting dyes into FIT probes is anticipated to be beneficial in a multitude of application scenarios.

## Author contributions

A. H. synthesized and evaluated the properties of oligonucleotide probes. A. K. and U. G. performed the biological experiments. A. H. wrote the first draft of the manuscript. O. S. conceived the research and wrote the final draft of the manuscript.

## Conflicts of interest

There are no conflicts to declare.

## Supplementary Material

SC-016-D4SC06729K-s001

## Data Availability

The datasets supporting this article have been uploaded as part of the ESI.†
