# Supplementary material for: Light harvesting FIT DNA hybridization probes for brightness-enhanced RNA detection
Source: Chem Sci. 2024 Dec 2;16(2):846–53. doi: 10.1039/d4sc06729k (PMC11622247; doi:10.1039/d4sc06729k)
Supplement: SC-016-D4SC06729K-s001 [file SC-016-D4SC06729K-s001.pdf]

Supplementary Information (SI) for Chemical Science.
This journal is © The Royal Society of Chemistry 2024

Light harvesting FIT DNA hybridization probes for brightness-enhanced RNA detection

Amal Homer, Andrea Knoll, Uschi Gruber and Oliver Seitz*

1. General procedures 2
2. DNA synthesis and labeling 4
3. Characterization for the probes 7
4. Optical properties, Tm, absorbance and fluorescence spectra 9
5. Spectral overlap for FRET 23
6. Förster-Radii 24
7. Sequences used for probe design 25
8. Cell work 25
9. Histograms from flow cytometry analyses 28
10. UPLC traces and MALDI-TOF-MS 31
11. References 45

1. General Procedures

General Information

Reagents were purchased from *Sigma Aldrich* (St. Louis, United States), *Alfa Aesar* (Ward Hill, United States), *ACROS Organics* (Geel, Belgium) and *Carbolutions* (St. Ingbert, Germany). Alexa Fluor 568 azide and Alexa Fluor 488 azide dyes were purchased from Lumiprobe GmbH (Hannover, Germany). 5'-Dimethoxytrityl-N4-benzoyl-5-(octa-1,7-diynyl)-2'-deoxycytidine,3'-[(2-cyanoethyl) -(N,N-diisopropyl)]-phosphoramidite (C8-Alkyne-dC-CE Phosphoramidite) was purchased from Glen Research (Davis Drive, Sterling, United States). Synthetic oligoribonucleotides were obtained from Biomers.net GmbH (Ulm, Germany). TLC plates, Silica gel 60 F254 were obtained from Merck (Darmstadt, Germany). DMF, and $CHCl_3$ were dried using solvent purification system *SPS 800* of *MBraun* (Garching, Germany). Buffer solutions were made of water purified by Astacus ultra purification system from *membraPure* (Henningsdorf, Germany).

Preparative HPLC

Oligonucleotide probes were purified by using a *1105 HPLC system* from *Gilson* (Limburg, Germany) equipped with a *X-Bridge BEH 130* C18 (10x150mm, 5 µm) from Waters (Milford, MA, USA), which was heated to 55 °C. Binary mixtures of A (triethyl ammonium acetate buffer pH: 7) and B (acetonitrile) were used as mobile phase in a linear gradient at a flow rate of 7ml/min.

Analytical HPLC

Probes were analyzed on the Waters Aquity H-Class system (Milford, MA, USA) equipped with a QDa detector. Alternatively, probes were also analyzed with an Agilent 1260 Infinity II HPLC system (Santa Clara, CA, USA) with an Agilent 1260 Infinity II diode array detector. Analysis was performed using Acquity UPLC Oligonucleotide BEH C18 Columns, (50/2.1 mm; 1.7µM; 130Å) at 50°C in both the cases and binary mixture of A (0.1M triethylammonium acetate pH: 7.5) and B (acetonitrile) was used.

Gradient I: 3%B to 30%B in 4min, 0.5ml/min for Waters analytical HPLC

Gradient II: 3%B to 40%B in 7min, 0.5ml/min for Agilent analytical HPLC

MALDI-TOF mass spectrometry

Mass spectra were recorded using a Shimadzu Axima Confidence mass spectrometer (Kyoto, Japan). To prepare the samples, a solution containing 50 mg/ml 3-hydroxypicolinic acid in acetonitrile/water (1:1 v/v) was combined with an equal solution of diammonium hydrogen citrate (50 mg/ml, 1:1 v/v). The matrix and analyte were co-crystallized using the dried droplet method: 1.0 µl of matrix solution and 0.8 µl of analyte solution were mixed on a 96-point ground steel MALDI target and then dried at room temperature. Samples were measured in positive mode with an ion gate of 5000 Da and pulsed extraction adjusted to the respective analyte mass. The laser was operated at 50 Hz and a power of 90-120. Between 200 and 600 single shots were accumulated per mass spectrum. The recorded spectra were calibrated with the single protonated ion signals of the oligonucleotide calibration standard (Bruker Daltonics, Bremen, Germany).

UV-Vis spectroscopy

The UV-Vis absorption spectra of the probes were measured using the V-750 spectrophotometer equipped with the PAC-743R Peltier cell changer (Jasco, Tokyo, Japan) and the F250 cooling unit (Julabo, Seelbach, Germany). Absorbance was recorded in steps of 1 nm over the range of 800-220 nm using quartz cuvettes (10 mm, 1.4 ml).

Probe concentration: The absorbance spectra of the probe solution (800-220 nm) were recorded, and the probe concentration was determined by analyzing the absorbance at 260 nm using the Beer-Lambert law. The molar extinction coefficients of the oligonucleotide sequences were calculated using the OligoAnalyzer (Integrated DNA Technologies, Coralville, IA, USA).[28]

Melting temperature: dissolved in phosphate buffer (10 mM Na_2HPO_4, 100 mM NaCl, pH 7) at a concentration of 0.2 µM, both the FIT probe and the complementary RNA strand were recorded at 260 nm from 20 to 90 °C at a heating rate of 0.5 °C/min in quartz cuvettes (10 mm, 1.4 ml). Each experiment was performed in triplicate and the resulting melting curves were smoothed, normalized, and melting temperature, Tm calculated as the maximum of the first derivative.

Fluorescence Spectroscopy

Fluorescence spectra were acquired using a Varian Carry Eclipse fluorescence spectrometer (Agilent Technologies, Santa Clara, CA, USA) and 10 mm quartz cuvettes. FIT probes and, if included, target RNA was dissolved in phosphate buffer (10 mM Na2HPO4, 100 mM NaCl, pH: 7) at specified ratios of 1:1 and 1:4. Experimental settings included λ_{ex} (excitation wavelength) at 560 nm, λ_{em} (emission wavelength) at 570-800 nm for QB-FIT probes and λ_{ex}: 485nm, λ_{em}: 495-700nm for TO-FIT probes with $slit_{ex}$ (excitation slit width) at 5 nm, and $slit_{em}$ (emission slit width) at 5 nm. Spectra were averaged over three scans, and samples were allowed to equilibrate for 2 minutes before each measurement. Fluorescence spectra were recorded at both 25 and 37 °C. The ATTO 590 dye, was utilized for determining relative quantum yields for QB FIT probes and ATTO530 was used for TO-FIT probes, procured from ATTO tech GmbH (Siegen, Germany). Comparisons of independent replicates indicated that fluorescence measurements exhibited reproducibility with a deviation of 5-10%.

Limit of detection measurements

A black 140 µl high-performance quartz glass cuvette (Hellma, Müllheim, Germany) was loaded with either PBS buffer (10 mM Na_2HPO_4, 100 mM NaCl, pH 7.0) or 100% CCRF-CEM cell lysate, and an initial blank measurement was taken. Subsequently, the C7-2AF568-QB-OMe probe or C7-QB-OMe probe was added. Increasing concentrations of RNA target were then added step by step, with each addition followed by incubation at 37 °C for 15 minutes before fluorescence measurement. Following the addition of each component, fluorescence measurements were carried out. QB fluorescence (λ_{ex} = 560 nm, λ_{em} = 570–800 nm, readout at 600 nm, $slit_{ex}$: 5nm, $slit_{em}$: 5nm) was recorded sequentially at 37 °C.

2. DNA synthesis and labeling

Automated DNA synthesis:

Oligonucleotides containing a serinol (Ser) nucleotide (synthesis of the N-Tfa-protected serinol building block has been described elsewhere), C8-Alkyne-dU-CE Phosphoramidite (Synthesized according to a previous report) [29] and C8-Alkyne-dC-CE Phosphoramidite (purchased from Glen Research, Davis Drive, Sterling, United States) were synthesized using automated DNA synthesis

on the Mermade 4 bioautomation (Irving, TX, USA).[21, 29] CPG carriers (1 µmol, pore size 500 Å) and DNA/LNA phosphoramidites and 2'-O-Me-building blocks were purchased from Link Technologies (Bellshill, UK). All phosphoramidites were used according to the manufacturer's instructions. DNA synthesis reagents were purchased from Carl Roth (Karlsruhe, Germany) and EMP-Biotech (Berlin, Germany). Each coupling step was monitored by measuring the absorbance of the DMT cleavage solutions. Oligonucleotide synthesis was performed at a scale of 1 µM and programmed to retain the DMT group at the end of the sequence. After synthesis, the CPG-supported oligonucleotides were dried under reduced pressure and transferred to 2-ml tubes. To each tube, 1 ml of aqueous ammonia (35%) was added and the mixture was shaken at 55 °C for 2 hours. The supernatant was collected and the volatile components were removed under reduced pressure. The crude product was purified by preparative RP-HPLC (DMT-ON- 15-40% B in A for 15 min; A = 0.1 M triethylammonium acetate pH 7.5, B = MeCN). Subsequently, the DMT group was removed by treatment with 50% AcOH for 1 h at room temperature. The oligonucleotides were precipitated in isopropanol and, if necessary, purified by preparative RP-HPLC (DMT-Off-5-40% B in A for 15 min. A = 0.1 M triethylammonium acetate pH 7.5, B = MeCN). Finally, the oligonucleotides were precipitated by adding 1 ml isopropanol containing 10 vol% aqueous NaCl (1M), cooled at -20 °C for 2 hours and centrifuged. The supernatant was removed, the pellet was dried under reduced pressure and resuspended in 200 µl water before characterization by UPLC™ UV-Vis spectroscopy and MALDI-TOF mass spectroscopy.

Conjugation of Alexa Fluor 568/Alexa Fluor 488 dyes

Alexa 568/Alexa 488 azide was conjugated to the alkyne-modified oligonucleotide probes through a Cu-Click reaction using a modified protocol from ATTO-Tech GmbH.[30] Here is a detailed description of the procedure:

Oligonucleotide Preparation:

- 10 nmol of alkyne-modified oligonucleotide was taken in a 500 µl screw cap tube and lyophilized.

Preparation of Stock Solutions:

- Prepare a 50 mM stock solution of Alexa Fluor 568/488 azide in DMSO (Solution A).

- Prepare 0.1 M solutions of THPTA ligand (Solution B) and CuSO4 (Solution C) in Milli-Q water.
- Mix Solutions B and C in a 2:1 ratio and agitate for 30 minutes at room temperature to form the Cu-THPTA complex (Solution D).
- Prepare a 0.2 M sodium ascorbate solution (Solution E) in Milli-Q water.

Cu-Click Reaction:

- Mix 1 µl of Solution E with 3 µl of Solution D. Observe the color change from deep blue to colorless, indicating the formation of the active Cu(I) complex.
- Add an additional 1 µl of Milli-Q water to this solution, making the total volume 5 µl.
- Add 1 µl of Solution A to the lyophilized oligonucleotide and an additional 4 µl of DMSO (Condition for single coupling. For double coupling, 3 µl of solution A and 2 µl DMSO was added)
- Add the 5 µl of the active Cu(I) solution prepared and incubate at 45 °C for 3 hours.

Quenching and Precipitation:

- After the reaction, add 100 µl of 3M NH_4OAC solution.
- Transfer the entire reaction mixture into a 2 ml screw cap tube.
- Add 1.5 ml of isopropanol, cool at -20 °C for 2 hours, and centrifuge at 14,000 rpm for 10 minutes.
- Resuspend the obtained pellets in water, remove insoluble materials by filtration through a 0.2 µm nylon syringe filter.

Purification:

- Purify the Alexa Fluor 568/488 probes with RP-HPLC. Combine product fractions, lyophilize, and desalt by precipitation (NaCl/isopropanol).

Conjugation of QB, TO and TO_{tric} dyes

QB, TO and TO_{tric} were introduced into the probe sequence using a method previously described for AF 568-free and AF 568-coupled oligos.[21]

3. Characterization of probes

Table S1: Characterization date for all the probes

Probe	Sequence 5' – 3'	MALDI-TOF		R_t [min]
		calc. $[M+H]^+$	Found $[M+H]^+$	
C1-QB	ATACTGCGTATC$\mathbf{U}_{alk}\mathbf{S}_{QB}T_L$CCCAAGGCTG<br>CT	8494	8494	2.29 [a]
C2-QB	ATACTGCGTA$\mathbf{U}_{alk}$CT$\mathbf{S}_{QB}$T$_L$CCCAAGGCTG<br>CT	8494	8497	2.18 [a]
C3-QB	ATACTGCG$\mathbf{U}_{alk}$ATCT$\mathbf{S}_{QB}$T$_L$CCCAAGGCTG<br>CT	8494	8494	2.11 [a]
C4-QB	ATAC$\mathbf{U}_{alk}$GCGTATCT$\mathbf{S}_{QB}$T$_L$CCCAAGGCTG<br>CT	8494	8493	2.08 [a]
C1-AF568	ATACTGCGTATC$\mathbf{U}_{AF568}$**S**T$_L$CCCAAGGCTG<br>CT	8944	8951	1.86 [a]
C2-AF568	ATACTGCGTA$\mathbf{U}_{AF568}$CT**S**T$_L$CCCAAGGCTG<br>CT	8944	8950	1.84 [a]
C3-AF568	ATACTGCG$\mathbf{U}_{AF568}$ATCT**S**T$_L$CCCAAGGCTG<br>CT	8944	8949	1.82 [a]
C4-AF568	ATAC$\mathbf{U}_{AF568}$GCGTATCT**S**T$_L$CCCAAGGCTG<br>CT	8944	8946	1.84 [a]
C1-AF568-QB	ATACTGCGTATC$\mathbf{U}_{AF568}\mathbf{S}_{QB}T_L$CCCAAGGC<br>TGCT	9268	9274	2.15 [a]
C2-AF568-QB	ATACTGCGTA$\mathbf{U}_{AF568}$CT$\mathbf{S}_{QB}$T$_L$CCCAAGGC<br>TGCT	9268	9270	2.12 [a]
C3-AF568-QB	ATACTGCG$\mathbf{U}_{AF568}$ATCT$\mathbf{S}_{QB}$T$_L$CCCAAGGC<br>TGCT	9268	9275	2.13 [a]
C4-AF568-QB	ATAC$\mathbf{U}_{AF568}$GCGTATCT$\mathbf{S}_{QB}$T$_L$CCCAAGGC<br>TGCT	9268	9274	2.12 [a]
C5-2AF568-QB	ATACTGCGTATC$\mathbf{U}_{AF568}\mathbf{S}_{QB}\mathbf{U}_{AF568}$CCCAAG<br>GCTGCT	10104	10102	3.62 [b]
C6-2AF568-QB	ATACTGCGTATC $\mathbf{U}_{AF568}\mathbf{S}_{QB}T_L$CCCAAGGC<br>$\mathbf{U}_{AF568}$GCT	10132	10130	3.51 [b]
C7-2AF568-QB	ATAC$\mathbf{U}_{AF568}$GCGTATC$\mathbf{U}_{AF568}\mathbf{S}_{QB}T_L$CCCAA<br>GGCTGCT	10132	10140	3.32 [b]
J1-2AF568-QB	GGTG$\mathbf{U}_{AF568}$AGCCATA$\mathbf{S}_{QB}$T$_L\mathbf{U}_{AF568}$AGCCG<br>AACAGGUC	10560	10557	3.52 [b]
C5-2AF568	ATACTGCGTATC$\mathbf{U}_{AF568}$**S**$\mathbf{U}_{AF568}$CCCAAGG<br>CTGCT	9778	9778	3.24 [b]
C6-2AF568	ATACTGCGTATC$\mathbf{U}_{AF568}$**S**T$_L$CCCAAGGC<br>$\mathbf{U}_{AF568}$GCT	9807	9801	3.38 [b]
C7-2AF568	ATAC$\mathbf{U}_{AF568}$GCGTATC$\mathbf{U}_{AF568}$**S**T$_L$CCCAAGG<br>CTGCT	9807	9805	3.09 [b]
J1-2AF568	GGTG$\mathbf{U}_{AF568}$AGCCATA**S**T$_L\mathbf{U}_{AF568}$AGCCGA<br>ACAGGUC	10235	10241	3.31 [b]

C7-QB-OMe	AUACU$_{alk}$GCGUAUCU$_{alk}$S$_{QB}$T$_{L}$CCCAAGGC UGCU	9143	9143	3.75[b]
C1-AF568-QB-OMe	AUACUGCGUAUCU$_{AF568}$S$_{QB}$T$_{L}$CCCAAGG CUGCU	9845	9851	3.29[b]
C6-2AF568-QB-OMe	AUACUGCGUAUCU$_{AF568}$S$_{QB}$T$_{L}$CCCAAGG C U$_{AF568}$GCU	10693	10695	3.60[b]
C7-2AF568-QB-OMe	AUACU$_{AF568}$GCGUAUCU$_{AF568}$S$_{QB}$T$_{L}$CCCAA GGCUGCU	10693	10700	3.43[b]
C8-2AF568-QB-OMe	AUACUGCGUAUCUGU$_{AF568}$CS$_{QB}$C$_{L}$AAGG CU$_{AF568}$GCT	10687	10687	2.53[a]
C9-2AF568-QB-OMe	AUACU$_{AF568}$S$_{QB}$C$_{L}$GUAUCUGU$_{AF568}$CCCAA GGGCUGCT	10677	10679	2.58[a]
C10-2AF568-QB-OMe	AU$_{AF568}$ACUGC$_{L}$S$_{QB}$U$_{AF568}$AUCUGUCCCAA GGCUGCT	10677	10683	2.83[a]
C11-2AF568-QB-OMe	AUACUGCGUAUCUGU$_{AF568}$S$_{QB}$C$_{L}$CAAGG C U$_{AF568}$GCT	10717	10720	2.54[a]
C12-2AF568-QB-OMe	CUCGAGCACU$_{AF568}$GS$_{QB}$C$_{L}$AGCCGGG U$_{AF568}$GCCUGG	10781	10785	2.39[a]
C13-2AF568-QB-OMe	GGUCCUCGAGCAC$_{AF568}$TS$_{QB}$T$_{L}$CAGCC$_{AF568}$GG GUGC	10758	10761	2.36[a]
C14-2AF568-QB-OMe	GGUC$_{AF568}$CUCGAGCAC$_{AF568}$TS$_{QB}$T$_{L}$CAGCCGG GUGC	10758	10757	2.39[a]
C15-2AF568-QB-OMe	UC$_{AF568}$AGCCGGGUGC$_{AF568}$S$_{QB}$T$_{L}$GGGCCA AAAUACT	10820	10830	2.88[a]
JK1-2AF568-QB-OMe	GGUGU$_{AF568}$AGCCAUAS$_{QB}$T$_{L}$U$_{AF568}$AGCCG AACAGGT	10843	10850	2.55[a]
JK2-2AF568-QB-OMe	GGU$_{AF568}$GUAGCCAU$_{AF568}$AS$_{QB}$T$_{L}$TAGCCG AACAGGT	10843	10849	2.63[a]
JK3-2AF568-QB-OMe	GGU$_{AF568}$GUAGCCAUAS$_{QB}$T$_{L}$U$_{AF568}$AGCCG AACAGGT	10857	10854	2.62[a]
JK4-2AF568-QB-OMe	GGUGU$_{AF568}$AGCCATA$_{L}$S$_{QB}$U$_{AF568}$TAGCCG AACAGGT	10856	10856	2.53[a]
C1-AF488-TO-OMe	AUACUGCGUAUCU$_{AF488}$S$_{TO}$T$_{L}$CCCAAGG CUGCU	9689	9696	2.20[a]
C1-AF488-TO$_{tric}$-OMe	AUACUGCGUAUCU$_{AF488}$S$_{TOtric}$T$_{L}$CCCAAG GCUGCU	9715	9711	2.34[a]
C6-2AF488-TO-OMe	AUACUGCGUAUCU$_{AF488}$S$_{TO}$T$_{L}$CCCAAGG C U$_{AF488}$GCU	10374	10377	2.18[a]
C6-2AF488-TO$_{tric}$-OMe	AUACUGCGUAUCU$_{AF488}$S$_{TOtric}$T$_{L}$CCCAAG GC U$_{AF488}$GCU	10399	10394	2.22[a]
C7-2AF488-TO -OMe	AUACU$_{AF488}$GCGUAUCU$_{AF488}$S$_{TO}$T$_{L}$CCCAA GGCUGCU	10374	10373	2.21[a]
C7- AF488-TO$_{tric}$ -OMe	AUACU$_{AF488}$GCGUAUCU$_{AF488}$S$_{TOtric}$T$_{L}$CCCA AGGCUGCU	10399	10407	2.33[a]
JK1-2AF488-TO -OMe	GGUGU$_{AF488}$AGCCAUAS$_{TO}$T$_{L}$U$_{AF488}$AGCCG AACAGGT	10524	10521	2.17[a]
JK1- AF488-TO$_{tric}$ -OMe	GGUGU$_{AF488}$AGCCAUAS$_{TOtric}$T$_{L}$U$_{AF488}$AGC CGAACAGGT	10549	10546	2.21[a]

[a] Waters analytical HPLC-RP-UV with gradient I [b] Agilent analytical HPLC-RP-UV with gradient I
(S): Serinol nucleotide

4. Optical properties, T_m, absorbance and fluorescence spectra

QB-FIT probes, AF568 probes and AF568-QB FIT probes

Table S2: Fluorescence intensities before and after hybridization and T_m values of C-QB-FIT probes, C-AF568 probes and C-AF568-QB FIT probes.

Probe	F_{ss} (600)	F_{ds} (600)	F_{ds}/F_{ss}	T_m/°C
C1-QB	1.6	97.5	59.8	73.8
C1-AF568	205.5	191.4	0.9	62.7
C1-AF568-QB	14.7	211.8	14.4	67.8
C2-QB	1.62	81.48	55.7	73.8
C2-AF568	195.4	199.3	1.0	61.2
C2-AF568-QB	19.1	197.0	10.3	67.4
C3-QB	1.70	105.9	58.1	74.0
C3-AF568	220.0	216.6	1.0	60.7
C3-AF568-QB	22.0	231.9	10.5	67.5
C4-QB	1.6	95.5	59.1	75.4
C4-AF568	179.8	275.2	1.5	60.1
C4-AF568-QB	37.6	291.7	7.7	70.2

Table S3: Optical properties of C-QB-FIT probes, C-AF568 probes and C-AF568-QB FIT probes (ϕ and maximum brightness, $Br_{max} = \varepsilon_{max} \cdot \phi_{ds}$)

Probe	ϕ_{ss}	ϕ_{ds}	ϕ_{ds}/ϕ_{ss}	$Br_{max}/10^3$
C1-QB	0.01 ± 0.001	0.53 ± 0.03	44.4 ± 1.3	67.1 ± 3.3
C1-AF568	1.01 ± 0.021	0.93 ± 0.01	0.9 ± 0.03	97.6 ± 3.7
C1-AF568-QB	0.05 ± 0.000	0.56 ± 0.02	12.1 ± 0.3	105.9 ± 1.1
C2-QB	0.01 ± 0.004	0.50 ± 0.03	43.9 ± 2.2	62.2 ± 2.4
C2-AF568	0.92 ± 0.037	0.99 ± 0.02	1.1 ± 0.1	103.1 ± 5.1
C2-AF568-QB	0.06 ± 0.002	0.55 ± 0.02	9.7 ± 0.6	103.6 ± 4.7
C3-QB	0.01 ± 0.005	0.52 ± 0.02	45.3 ± 3.5	65.9 ± 3.2
C3-AF568	1.00 ± 0.044	0.94 ± 0.02	0.9 ± 0.02	98.3 ± 5.5
C3-AF568-QB	0.06 ± 0.003	0.60 ± 0.01	9.6 ± 0.4	117.6 ± 2.6
C4-QB	0.01 ± 0.003	0.53 ± 0.01	44.5 ± 2.0	70.9 ± 3.6
C4-AF568	0.87 ± 0.033	0.95 ± 0.02	1.1 ± 01	106.5 ± 6.0
C4-AF568-QB	0.99 ± 0.004	0.65 ± 0.01	6.5 ± 0.2	129.6 ± 7.3

Probes targeted against complementary RNA (CCRF-CEM RNA: 5'-A-G-C-A-G-C-C-U-U-G-G-G-A-C-A-G-A-U-A-C-G-C-A-G-U-A-U-3'). F_{ss} and F_{ds} are fluorescence intensities measured before and after target hybridization at 600 nm. T_m is the melting temperature for the probe RNA duplex. ϕ_{ss} and ϕ_{ds} is the fluorescence quantum yield of single strand probe and probe-target duplex, respectively. Brightness (Br_{max}) = $\varepsilon_{max} \cdot \phi_{ds}$ in $M^{-1} \cdot cm^{-1}$. Conditions: 200 nM probe and 4eq of RNA target in PBS (100 mM NaCl, 10 mM Na_2HPO_4, pH 7) at 25 °C. Experiments were performed in triplicates.

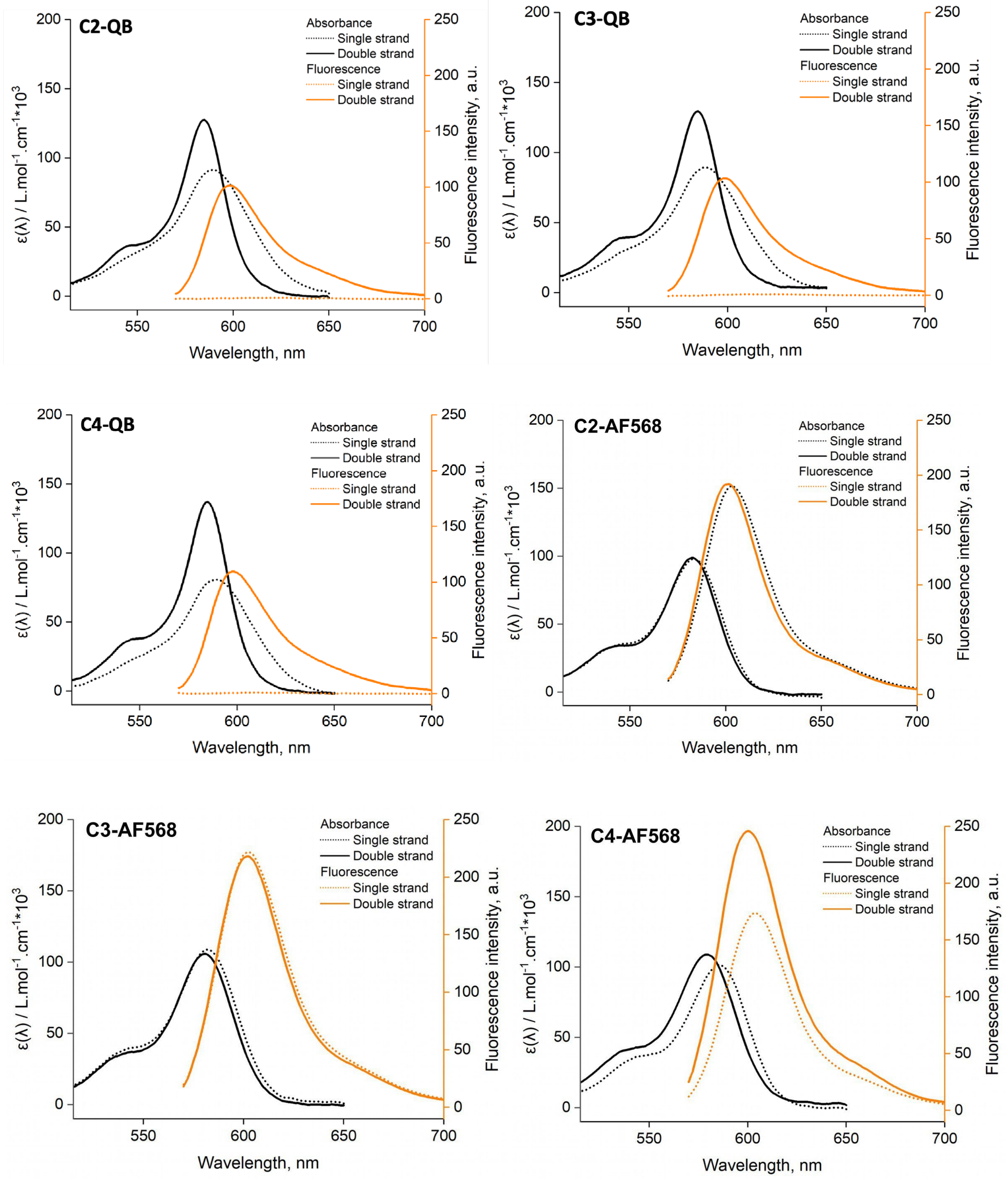

C2-QB
C3-QB
C4-QB
C2-AF568
C3-AF568
C4-AF568
Absorbance
Single strand
Double strand
Fluorescence
Single strand
Double strand
ε(λ) / L.mol-1.cm-1*10^3
Fluorescence intensity, a.u.
Wavelength, nm
0
50
100
150
200
250
550
600
650
700

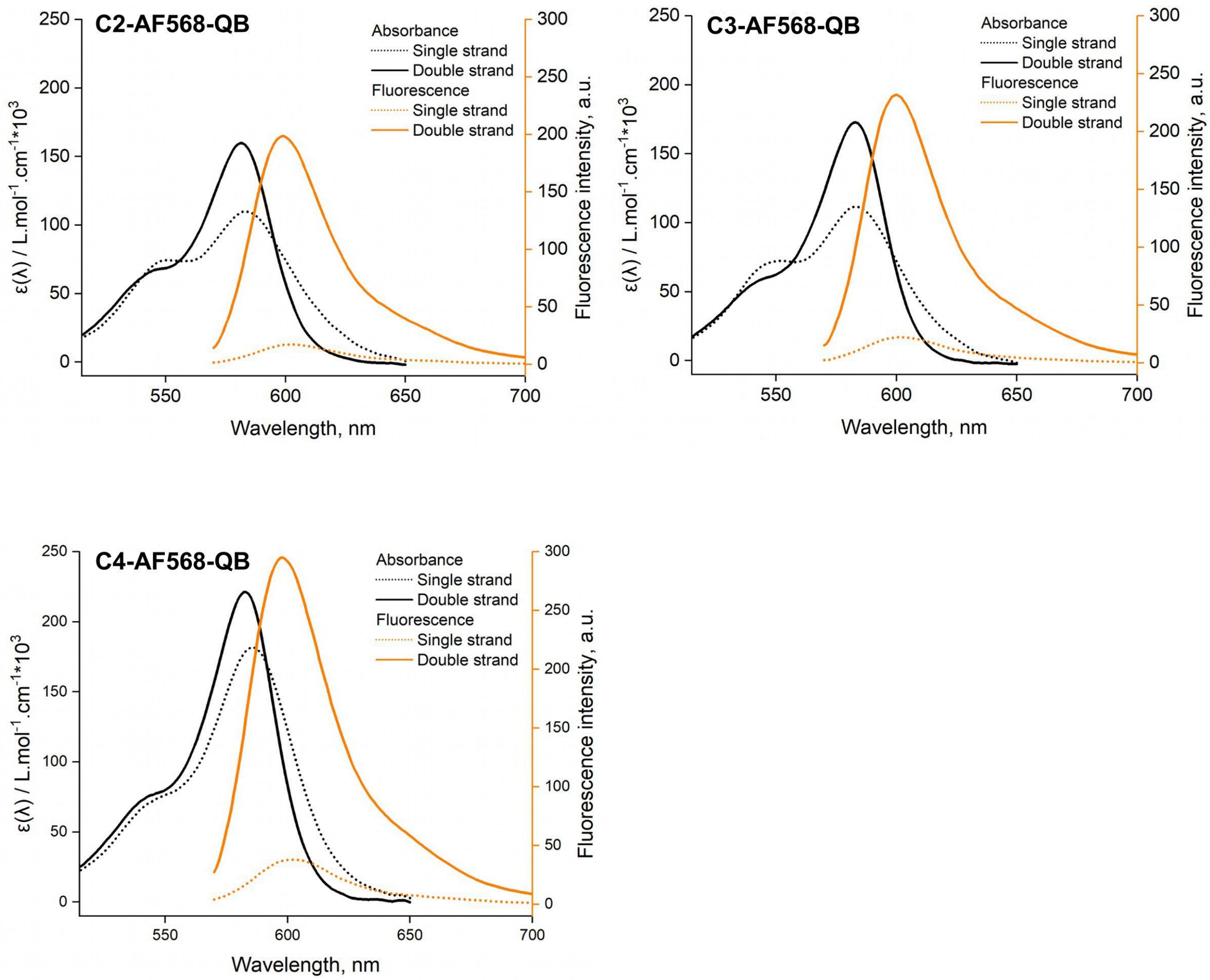

Figure S1: Absorbance and fluorescence spectra of the CCRF-CEM QB-FIT probes, AF568 probes, and AF568-QB FIT probes. Experimental conditions: 200 nM probe was incubated at 25 °C before and after the addition of 4 equivalents of RNA target. Excitation wavelength: 560 nm, Emission wavelength: 570-800 nm.

2AF568-probes and 2AF568-QB FIT probes

Table S4: Optical properties and T_m of probes targeted against the CDR3 region of the TCR mRNA from CCRF-CEM cells (C5-C7) or Jurkat cells (J1)

Probe	F_{ss}	F_{ds}	F_{ds}/F_{ss}	ϕ_{ss}	ϕ_{ds}	ϕ_{ds}/ϕ_{ss}	$Br_{max}/10^3$	T_m/°C
C5-2AF568	157.6	100.0	0.6	0.45	0.26	0.6	39.8	-
C5-2AF568-QB	12.4	22.2	1.8	0.02	0.04	1.7	7.7	66.0
C6-2AF568	295.8	388.4	1.3	0.76	0.84	1.1	162.1	-
C6-2AF568-QB	29.0	414.5	14.3	0.05	0.61	10.9	179.4	68.0
C7-2AF568	286.5	375.7	1.3	0.71	0.82	1.1	151.4	-
C7-2AF568-QB	24.5	413.6	16.9	0.04	0.59	12.6	177.4	70.4
J1-2AF568	354.7	385.3	1.1	0.84	0.81	1.0	167.0	-
J1-2AF568-QB	34.6	287.6	8.3	0.05	0.40	6.8	117.5	59.2

Probes targeted against complementary RNA (CCRF-CEM RNA: 5'-A-G-C-A-G-C-C-U-U-G-G-G-A-C-A-G-A-U-A-C-G-C-A-G-U-A-U-3', Jurkat RNA: 5'-A-C-C-U-G-U-U-C-G-G-C-U-A-A-C-U-A-U-G-G-C-U-A-C-A-C-C -3'). For details see caption to Table S3.

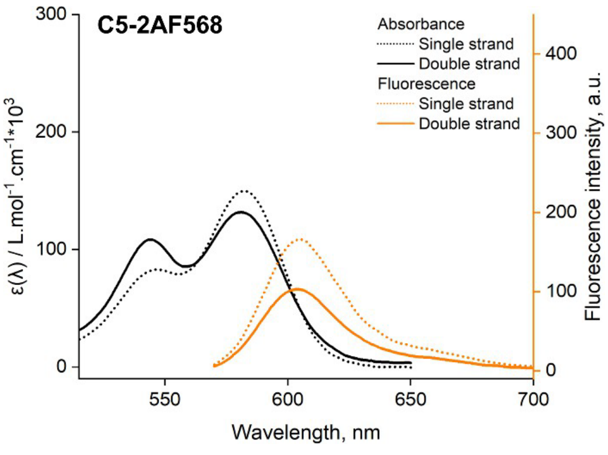

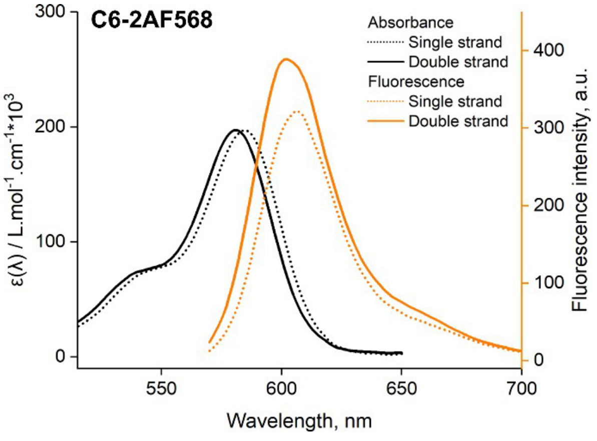

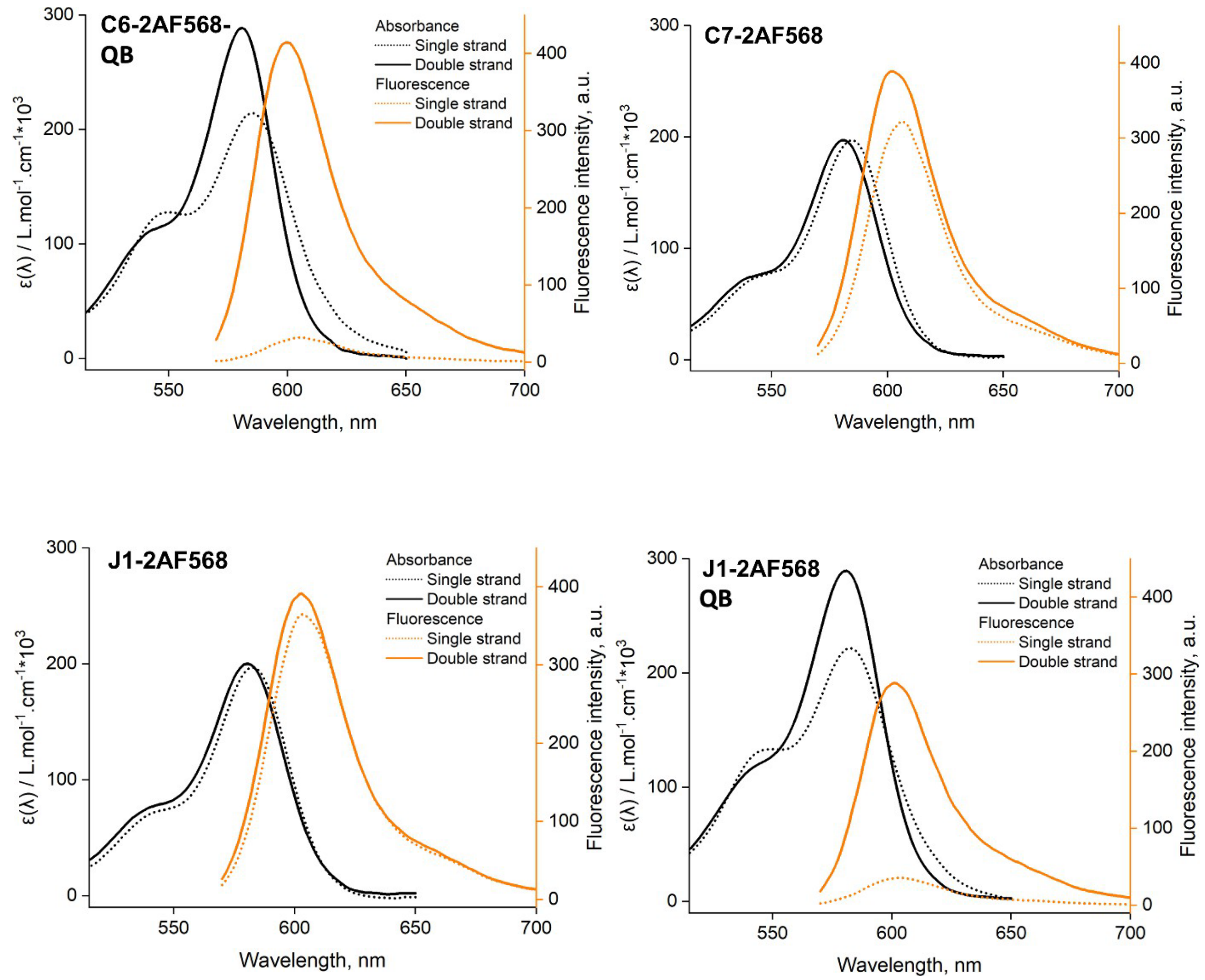

Figure S2: Absorbance and fluorescence spectra of the CCRF-CEM and Jurkat QB-FIT 2AF568 probes, and 2AF568-QB FIT probes. Experimental conditions: see caption to Fig. S1.

Table S5: Comparison between fluorescence properties of DNA FIT probes and 2'-OMe gapmer RNA FIT probes

**Probe**	**Sequence 5' – 3'**	F_{ss}	F_{ds}	F_{ds}/F_{ss}
C1-QB	ATACTGCGTATC**U**$_{alk}$**S**$_{QB}$T$_{L}$CCCAAGGCTGCT	1.6	97.5	59.8
C7-QB-OMe	AUAC**U**$_{alk}$GCGUAUC**U**$_{alk}$**S**$_{QB}$T$_{L}$CCCAAGGCUGCU	1.6	120.6	75.0
C1-AF568-QB	ATACTGCGTATC**U**$_{AF568}$**S**$_{QB}$T$_{L}$CCCAAGGCTGCT	14.7	211.8	14.4
C1-AF568-QB-OMe	AUACUGCGUAUC**U**$_{AF568}$**S**$_{QB}$T$_{L}$CCCAAGGCUGCU	13.0	250.0	19.3
C6-2AF568-QB	ATACTGCGTATC **U**$_{AF568}$**S**$_{QB}$T$_{L}$CCCAAGGC **U**$_{AF568}$GCT	29.0	414.5	14.3
C6-2AF568-QB-OMe	AUACUGCGUAUC**U**$_{AF568}$**S**$_{QB}$T$_{L}$CCCAAGGC **U**$_{AF568}$GCU	23.7	377.4	15.9
C7-2AF568-QB	ATAC**U**$_{AF568}$GCGTATC**U**$_{AF568}$**S**$_{QB}$T$_{L}$CCCAAGGCTGCT	24.5	413.6	16.9
C7-2AF568-QB-OMe	AUAC**U**$_{AF568}$GCGUAUC**U**$_{AF568}$**S**$_{QB}$T$_{L}$CCCAAGGCUGCU	28.3	512.5	18.1
J1-2AF568-QB	GGTG**U**$_{AF568}$AGCCATA**S**$_{QB}$T$_{L}$**U**$_{AF568}$AGCCGAACAGGUC	34.6	287.6	8.3
JK1-2AF568-QB-OMe	GGUG**U**$_{AF568}$AGCCAUA**S**$_{QB}$T$_{L}$**U**$_{AF568}$AGCCGAACAGGT	24.5	393.6	16.1

CCRF-CEM 2'-OMe gapmer FIT probes

Table S6: Optical properties of C-AF568-QB-OMe and C-2AF568-QB-OMe FIT probes (Fluorescence intensities F_{ss} and F_{ds} before and after hybridization, fluorescence quantum yields ϕ_{ss} and ϕ_{ds} before and after hybridization, maximum brightness $Br_{max} = \varepsilon_{max} \cdot \phi_{ds}$ and T_m)

Probe	F_{ss}	F_{ds}	F_{ds}/F_{ss}	ϕ_{ss}	ϕ_{ds}	ϕ_{ds}/ϕ_{ss}	$Br_{max}/10^3$	T_m/ °C
C7-QB-OMe	1.6	121	75.0	0.01	0.6	54.5	72.5	>90
C1-AF568-QB-OMe	13.0	250	19.3	0.03	0.52	14.4	109.6	>90
C6-2AF568-QB-OMe	23.7	377	15.9	0.04	0.6	11.5	174.3	>90
C7-2AF568-QB-OMe	28.3	513	18.1	0.05	0.64	13.3	224.8	>90
C8-2AF568-QB-OMe	27.5	326	11.8	0.06	0.54	9.2	135.0	78.7
C9-2AF568-QB-OMe	40.6	88	2.2	0.09	0.18	2.1	36.5	64.6
C10-2AF568-QB-OMe	32.9	138	4.2	0.06	0.23	3.7	56.8	87.4
C11-2AF568-QB-OMe	20.8	363	17.4	0.04	0.57	13.2	145.1	80.7
C12-2AF568-QB-OMe	36.9	322	8.7	0.06	0.49	7.2	135.1	>90
C13-2AF568-QB-OMe	73.6	286	3.9	0.15	0.5	3.3	130.3	>90
C14-2AF568-QB-OMe = CEM14	28.4	308	10.8	0.05	0.52	9.5	134.0	83.1
C15-2AF568-QB-OMe	42.8	311	7.3	0.08	0.51	6.0	134.2	83.1

Probes targeted against complementary RNA (CCRF-CEM, C1-C11 RNA: 5'-A-G-C-A-G-C-C-U-U-G-G-G-A-C-A-G-A-U-A-C-G-C-A-G-U-A-U and C12-C15 RNA: 5'-C-A-G-U-A-U-U-U-U-G-G-C-C-C-A-G-G-C-A-C-C-C-G-G-C-U-G-A-C-A-G-U-G-C-U-C-G-A-G-G-3'). For further details see caption to Table S3.

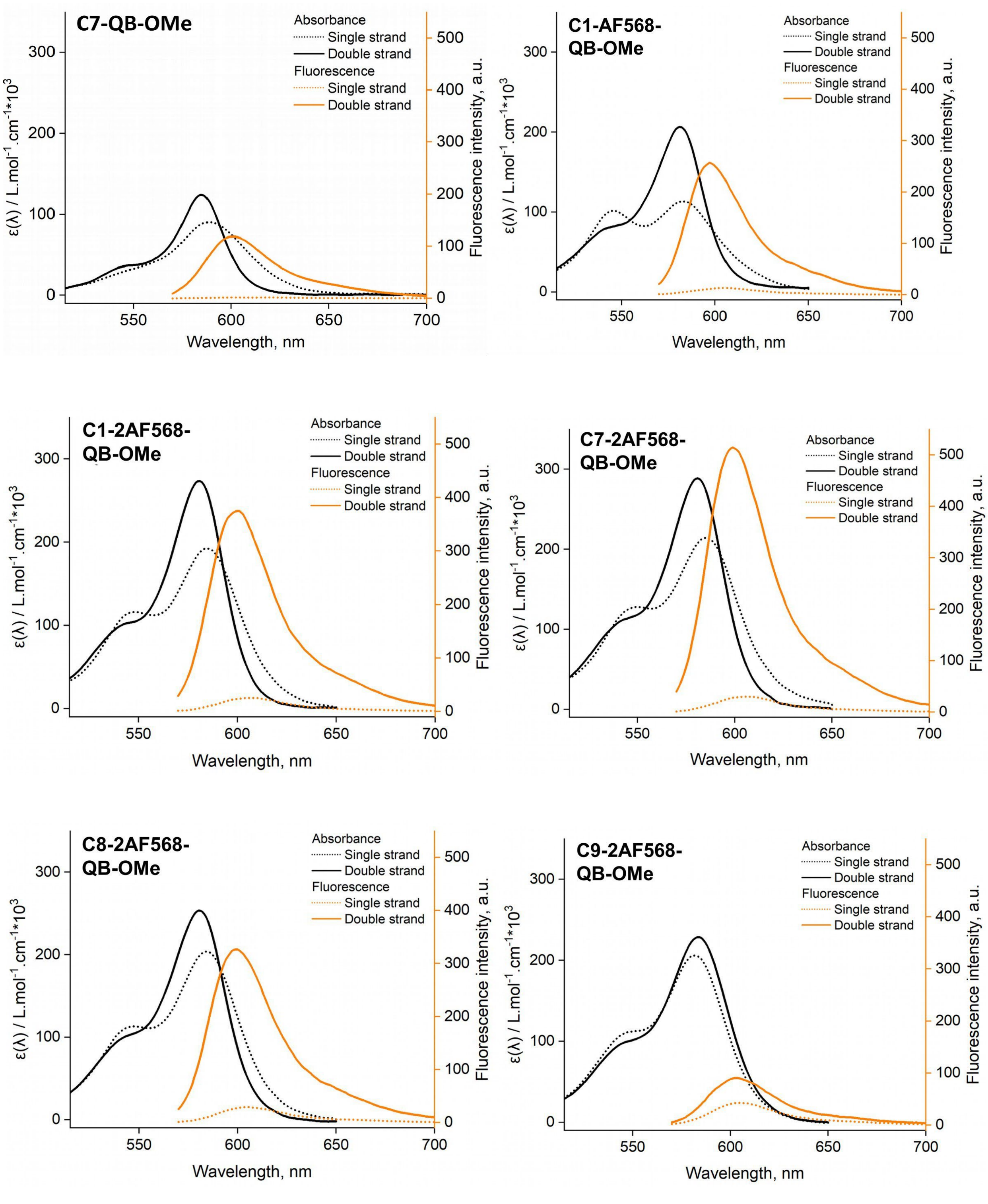
C7-QB-OMe
C1-AF568-QB-OMe
C1-2AF568-QB-OMe
C7-2AF568-QB-OMe
C8-2AF568-QB-OMe
C9-2AF568-QB-OMe
Absorbance
Single strand
Double strand
Fluorescence
Single strand
Double strand
ε(λ) / L.mol-1.cm-1*10^3
Fluorescence intensity, a.u.
Wavelength, nm

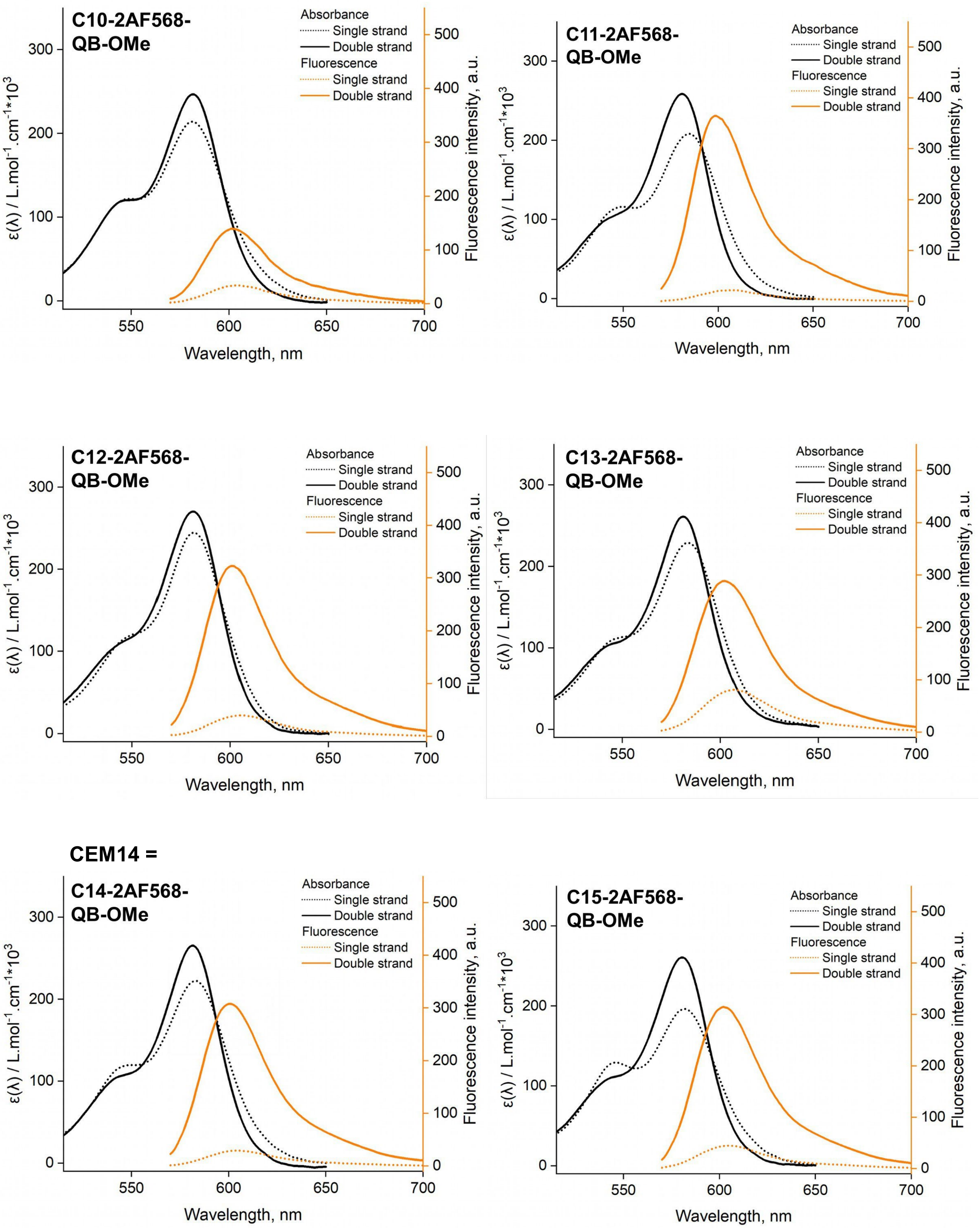

Figure S3: Absorbance and fluorescence spectra of the CCRF-CEM 2'-OMe 2AF568-QB FIT probes. Experimental conditions: see caption to Fig. S1.

Jurkat 2’-OMe gapmer FIT probes

Table S7: Optical properties of J-2AF568-QB FIT probes (Fluorescence intensity F_{ss}, F_{ds}, fluorescence quantum yields ϕ_{ss}, ϕ_{ds}, brightness $Br_{max} = \varepsilon_{max} \cdot \phi_{ds}$ and T_m)

Probe	F_{ss}	F_{ds}	F_{ds}/F_{ss}	ϕ_{ss}	ϕ_{ds}	ϕ_{ds}/ϕ_{ss}	$Br_{max}/10^3$	T_m/°C
J1-2AF568-QB-OMe	24.5	393.6	16.1	0.05	0.56	11.7	155.1	76.5
J2-2AF568-QB-OMe = JK2	23.4	157.9	6.7	0.05	0.25	5.3	65.3	78.9
J3-2AF568-QB-OMe	24.6	222.0	9.0	0.05	0.34	7.0	94.8	79.1
J4-2AF568-QB-OMe	21.1	160.3	7.6	0.04	0.26	6.0	62.9	74.0

Probes targeted against complementary RNA (Jukat RNA: 5'-A-C-C-U-G-U-U-C-G-G-C-U-A-A-C-U-A-U-G-G-C-U-A-C-A-C-C -3'). For further details: see caption to Table S3.

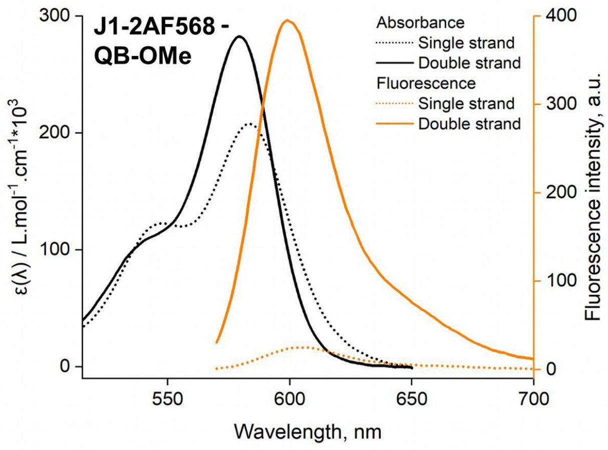

JK2 =

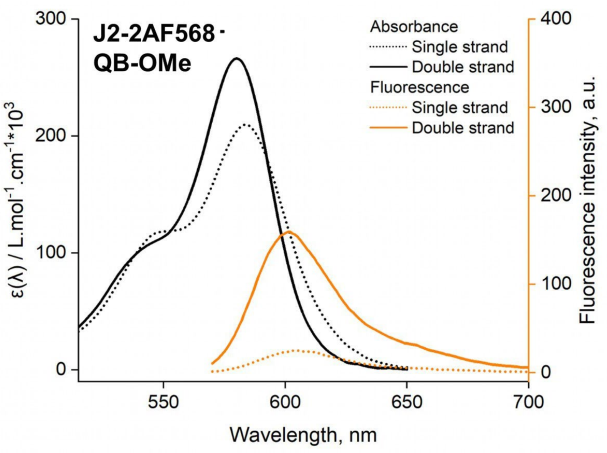

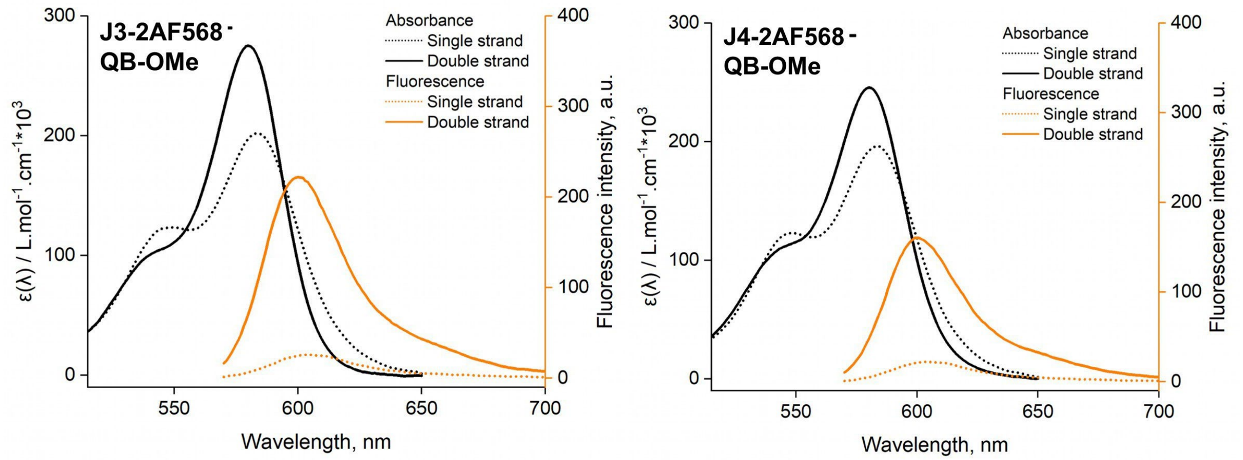

Figure S4: Absorbance and fluorescence spectra of the Jurkat 2'-OMe 2AF568-QB FIT probes. Experimental conditions: see caption to Fig. S1.

TO- and TO_{tric} 2'-OMe gapmer FIT probes

Table S8: Optical properties of TO and TO_{tric} FIT probes targeted against the CDR3 region of the TCR mRNA from CCRF-CEM cells (C1-C7) or Jurkat cells (JK1)

Probe	F_{ss}	F_{ds}	F_{ds}/F_{ss}	ϕ_{ss}	ϕ_{ds}	ϕ_{ds}/ϕ_{ss}	$Br_{max}/10^3$	T_m/°C
C1-AF488-TO-OMe	40.0	265.9	6.6	0.06	0.37	5.8	40.4	>90
C1-AF488-TO_{tric}-OMe	26.0	365.4	14.0	0.04	0.49	11.8	60.0	>90
C6-2AF488-TO-OMe	45.3	287.9	6.3	0.05	0.26	5.6	46.3	>90
C6-2AF488-TO_{tric}-OMe	45.1	284.5	6.3	0.05	0.31	5.9	52.0	>90
C7-2AF488-TO-OMe	54.8	500.1	9.1	0.05	0.37	7.9	77.0	>90
C7-2AF488-TO_{tric}-OMe	51.7	563.2	10.9	0.05	0.46	9.2	96.5	>90
JK1-2AF488-TO-OMe	52.3	471.8	9.0	0.04	0.36	8.2	82.3	80.0
JK1-2AF488-TO_{tric}-OMe	52.4	552.6	10.5	0.05	0.44	9.6	99.4	79.7

Probes targeted against complementary RNA (CCRF-CEM RNA: 5'-A-G-C-A-G-C-C-U-U-G-G-G-A-C-A-G-A-U-A-C-G-C-A-G-U-A-U-3', Jurkat RNA: 5'-A-C-C-U-G-U-U-C-G-G-C-U-A-A-C-U-A-U-G-G-C-U-A-C-A-C-C -3'). F_{ss} and F_{ds} , fluorescence intensities at 530 nm measured before and after target hybridization. T_m is the melting temperature for the probe RNA duplex. ϕ_{ss} and ϕ_{ds} are fluorescence quantum yield of single strand probe or probe-target duplex, respectively. $Br_{max} = \varepsilon_{max} \cdot \phi_{ds}$ in $M^{-1} \cdot cm^{-1}$. Conditions: 200 nM probe and 4 eq of RNA target in PBS (100 mM NaCl, 10 mM Na_2HPO_4, pH 7) at 25 °C.

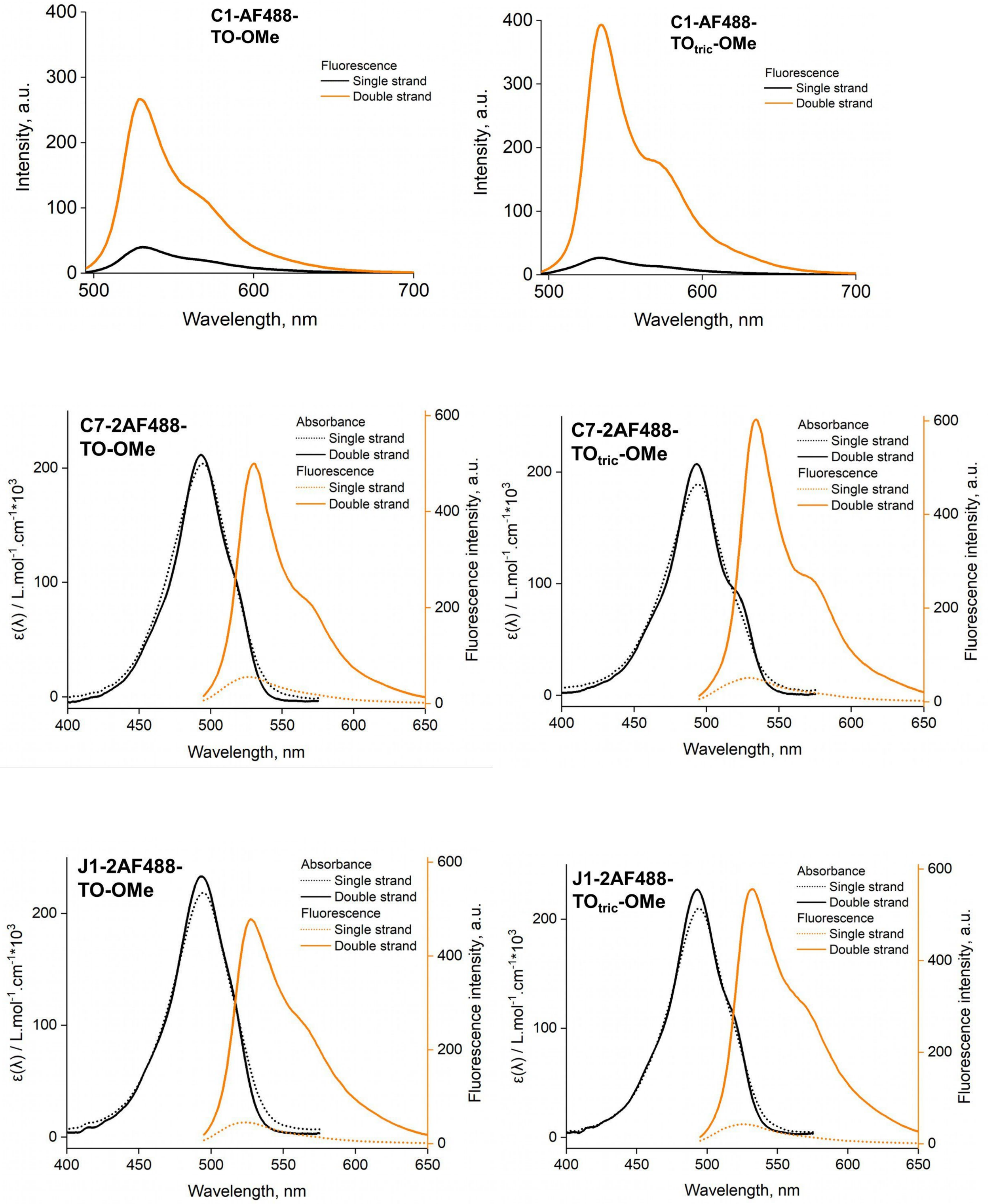

Figure S5: Absorbance and fluorescence spectra of the CCRF-CEM and Jurkat TO and TO$_{tric}$ 2'-OMe gapmer FIT probes. Experimental conditions: A 200 nM probe was incubated at 25 °C before and after the addition of 4 equivalents of RNA target. Excitation wavelength: 560 nm, Emission wavelength: 570-800 nm.

5. Spectral overlap for FRET

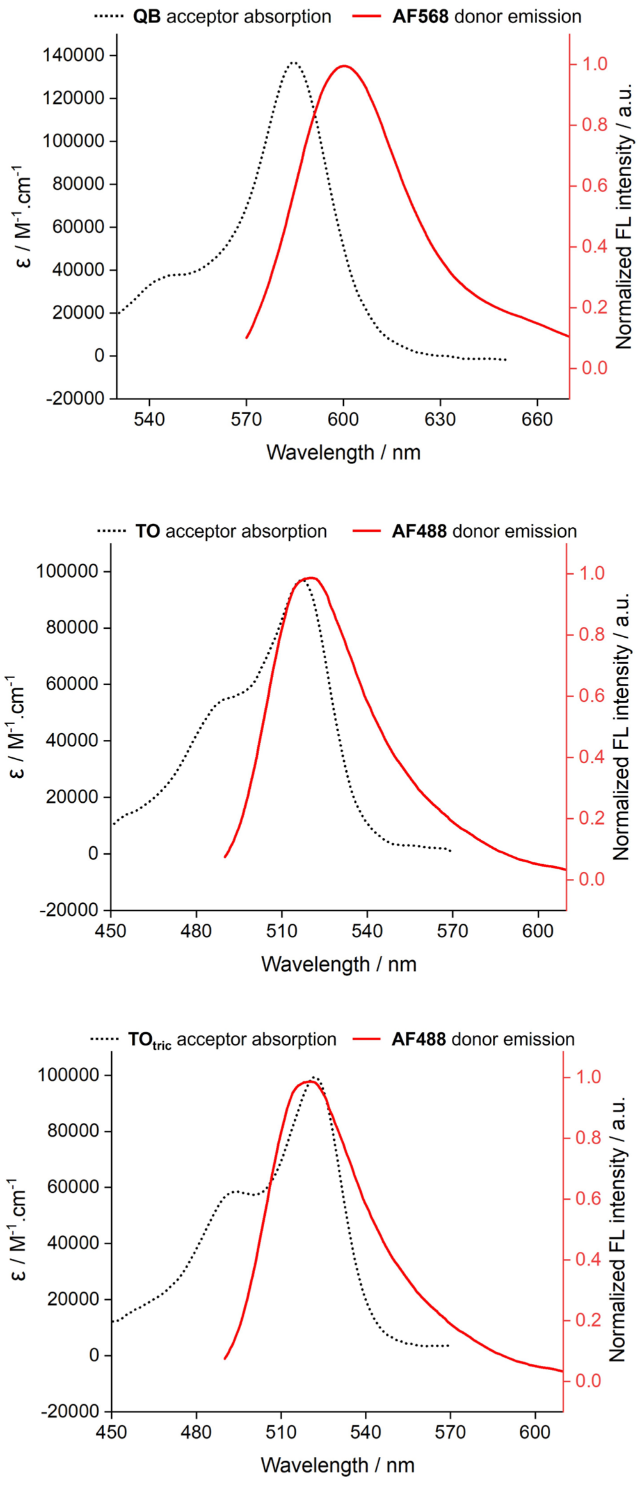

Figure S6: Absorption spectra for the acceptor dyes and normalized emission spectra for the donor dyes.

6. Förster radii

Table S9: Overlap integral (J(λ)) and Förster radii (R_0) for AF568-QB, AF488-TO and AF488-TO_{tric} FRET pairs in double stranded states of the light harvesting FIT probes.

probe	J(λ), $M^{-1}.cm^{-1}.nm^4$	R_0, Å (κ^2= 1/3)[a]	R_0, Å (κ^2= 2/3)[b]
C1-AF568-QB	4.86E+15	59.1	66.4
C2-AF568-QB	4.89E+15	59.5	66.8
C3-AF568-QB	5.11E+15	59.7	67.0
C4-AF568-QB	5.29E+15	59.9	67.2
C5-2AF568-QB	4.34E+15	46.8	52.6
C5-2AF568-QB	4.43E+15	57.1	64.1
C7-2AF568-QB	4.50E+15	57.0	64.0
C1-AF488-TO	3.38E+15	50.9	57.1
C1-AF488-TO_{tric}	3.78E+15	51.8	58.2

[a] κ^2= 1/3 if either the donor or the acceptor has a fixed orientation. [b] κ^2= 2/3 for a randomized orientation of donor and acceptor.

7. Sequences used for probe design

Targeted sequence from the CDR3 region of the TCRβ chain mRNA expressed in Jurkat cells

ACCTGTTCGGCTAACTATGGCTACACC

The sequence has been published by Yoshikai et al.[31]

Sequencing analysis was confirmed by HS Diagnomics by using forward primer binding sites (blue) and reverse primer binding sites (red)

TGAAGATCCAGCCCTCAGAACCCAGGGACTCAGCTGTGTACTTCTGTGCCAGCAGTT
TCTCGACCTGTTCGGCTAACTATGGCTACACCTTCGGTTCGGGGACCAGGTTAACCG
TTGTAGAGGACCTGAACAAGGTGTTCCCACCCGAGGTCGCTGTGTTT
GAGCCATCAGAAGCAGAGATCTCCCACAC

Targeted sequence from the CDR3 region of the TCRβ chain mRNA expressed in CCRF-CEM cells

Sequencing analysis was performed by HS Diagnomics by using forward primer binding sites (blue) and reverse primer binding sites (red) to identify a region (green) distinct from transcripts found in Jurkat cells

TCTCACCTAAATCTCCAGACAAAGCTCACTTAAATCTTCACATCAATTCCCTGGAGC
TTGGTGACTCTGCTGTGTATTTCTGTGCCAGCAGCCTTGGGACAGATACGCAGTATTT
TGGCCCAGGCACCCGGCTGACAGTGCTCGAGGACCTGAAAAACGTGTTCCCACCCG
AGGTCGCTGTGTTTGAGCCATCAGAAGCAGAGATCTCCCACAC

8. Cell work

Cell Culture

CCRF-CEM cells and Jurkat cells were cultured in RPMI supplemented with 10% FBS and 1% penicillin/streptomycin at 37°C and 5% CO_2. Cells were passaged twice a week by treating them with 0.1% trypsin/EDTA at 37°C for 3 min. Then the cells were centrifuged and resuspended in fresh medium. Cells from passage 1 to 6 after thawing from cryopreservation were used. Cell lines were originally purchased from the Leibniz Institute DSMZ-German Collection of Microorganisms and Cell Cultures: Jurkat (ACC 282) and CCRF-CM (ACC 240) and provided by Prof. Marcus Frohme (Technical University of Applied Sciences Wildau, Wildau, Germany)).

Preparation of cell lysate

CCRF CEM cells were pelleted by centrifugation (500 x g, 5 min), washed with PBS buffer, and lysed by 15 min incubation in 4 ml lysis buffer (10 mM Na_2HPO_4, 100 mM NaCl, 0.5 % Triton X-100, pH 7.0, 1 U/µl RNAsin Plus RNAse inhibitor [Promega, Madison, WI, USA], Complete

Mini protease inhibitor cocktail tablets [Sigma-Aldrich, St. Louis, MO, USA] 1 tbl/10 ml) inhibitors on ice for 20 min with sonification every 5 min. Cell debris and nuclei were removed by centrifugation (14000 x g, 10 min; 4 °C) and the supernatant was aliquoted and stored at −80 °C.

Probe delivery by electroporation

Cells were harvested, centrifuged, resuspended in PBS and counted with an automated cell counter (Countess II, Invitrogen, Waltham, MA, USA). One million cells per sample were pipetted in 1.5 mL Eppendorf tubes, centrifuged and resuspended in RPMI without glutamine and FBS. FIT probes were added in the specified concentration. The total volume of 200 µL was transferred to cuvettes for electroporation (gap width 4 mm, BioRad, Hercules, CA, USA).

Electroporation was performed at room temperature, using a Gene Pulser® Xcell™ electroporation system (BioRad) with the following pulse protocol: 1 pulse at 240 V for 25 ms. After adding of 800 µL RPMI, cells were incubated for 3 h at 37°C. Then cells were washed, resuspended in PBS and treated with SYTOX™ Red Dead Cell Stain (Invitrogen, Carlsbad, CA, USA) for 15 min.

Flow Cytometry

Flow cytometry was performed on a FACSMelody™ cell sorter (Beckton-Dickinson, Franklin Lakes, NJ, USA), equipped with a blue (488 nm), a yellow-green (561 nm) and a red (640 nm) laser and five mirror/filters pairs (2B-2R-2YG configuration): set A: 665LP, 700/54, set B: 507LP, 527/32, set C: 752LP, 783/56, set D: 660/10, 660/10, set E: 752LP, 783/56, set F: 665LP, 697/58, set G: 605LP, 613/18, set H: 582LP, 582/15, side scatter: ND10, 488/15. Drop delay was automatically adjusted with BD FACS™ Accudrop beads prior to each run. During measurement, samples were agitated at 100 rpm, and the stream speed was adjusted to produce an event rate of $\leq 500\ s^{-1}$at 20.000 events per sample. Data were analyzed using FlowJo v10.10 software (FlowJo LLC, Ashland, OR, USA). Gating was performed as shown in Figure S7 and always starts with data of control cells to define the fluorescence area of background.

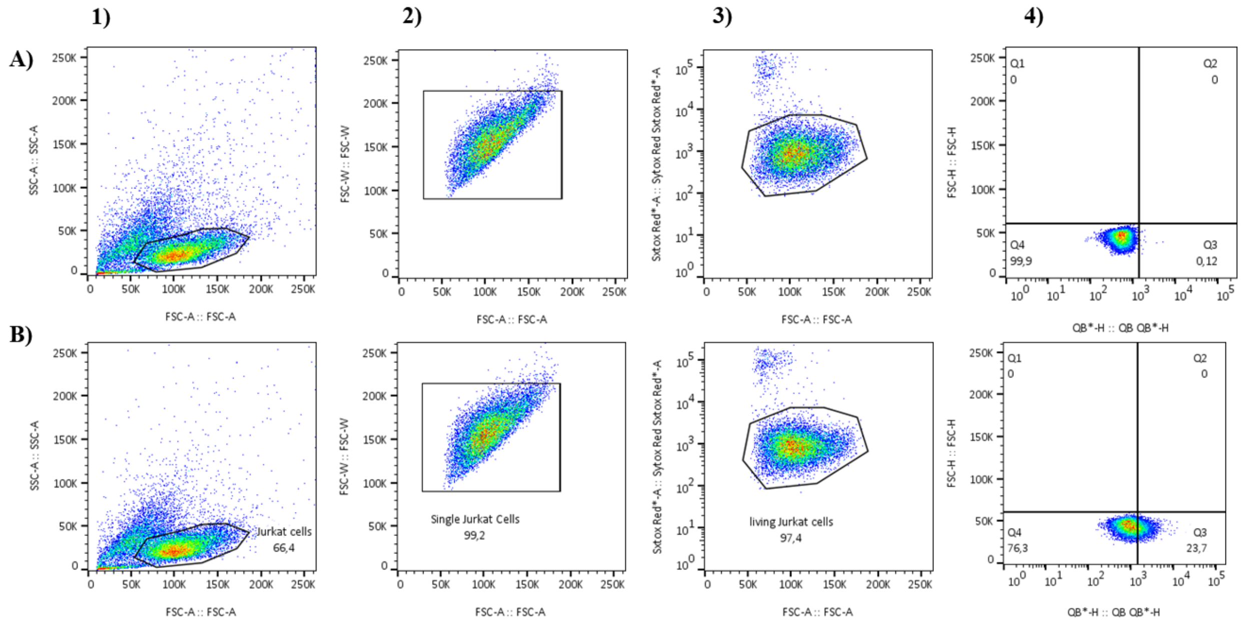

Figure S7: Example for gating by software FlowJo after flow cytometry. Scatter plots of Jurkat cells after electroporation in buffer A) without or B) with FIT probe. 1) Exclusion of debris from all events recorded, 2) gating single cells by exclusion of doublets, 3) exclusion of dead cells after staining with SYTOX™ Red, A4) application of quadrant tool to define background fluorescence, B4) application of the same quadrants to cells treated with FIT probe. Numbers in the diagrams refer to the percentage of gated cells within the corresponding cell population. Cells in Q3 show enhanced fluorescence in comparison to control cells.

9. Histograms from flow cytometry analyses

CEM14, 10 nM in electroporation buffer

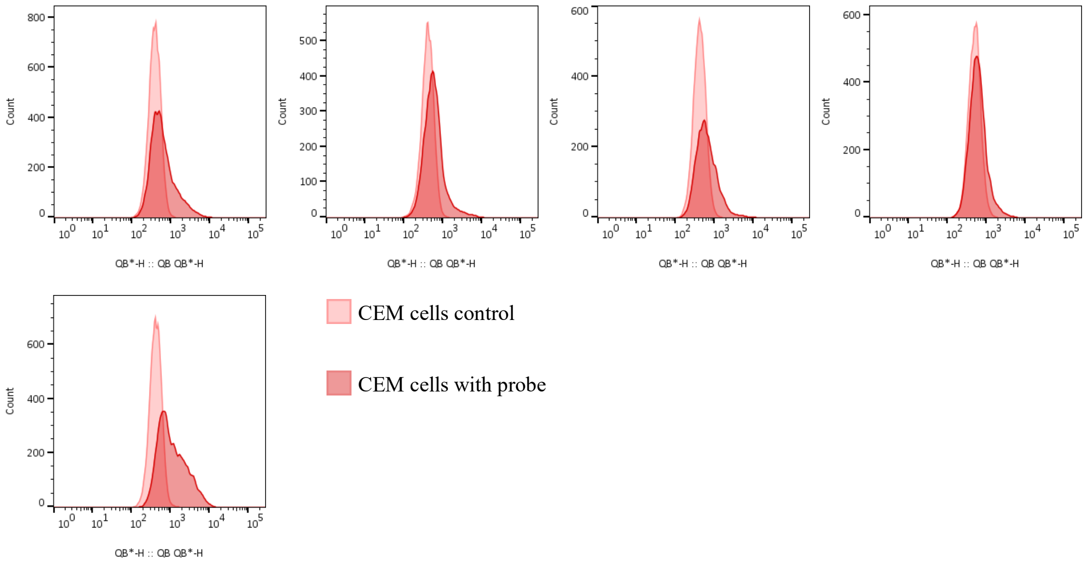

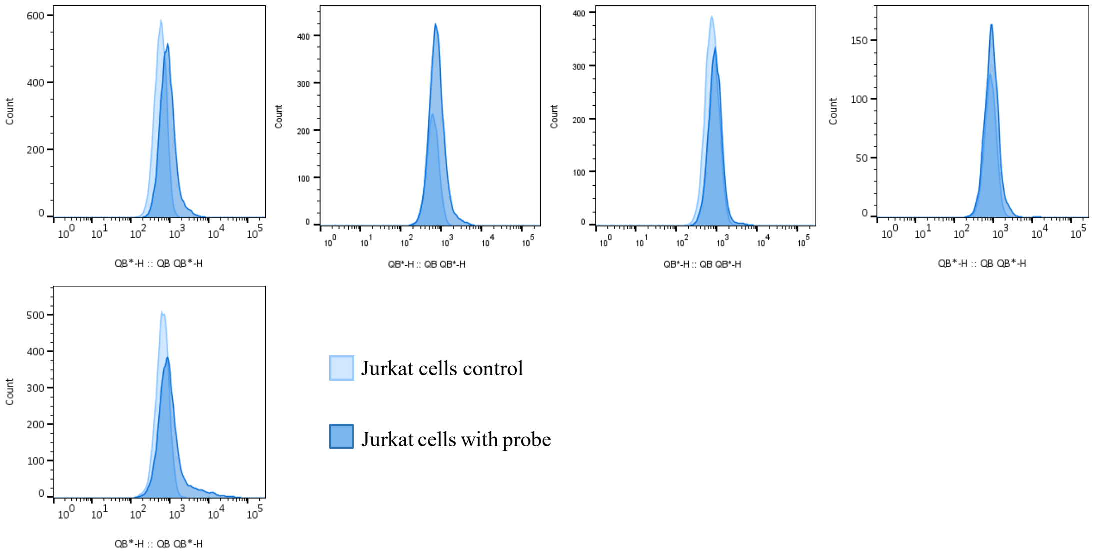

JK2, 100 nM in electroporation buffer

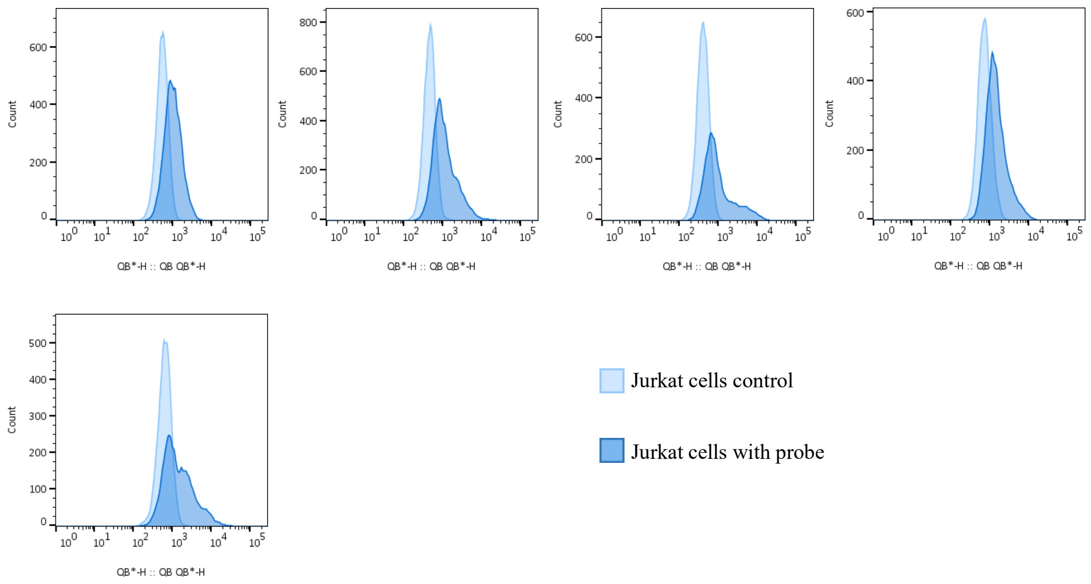

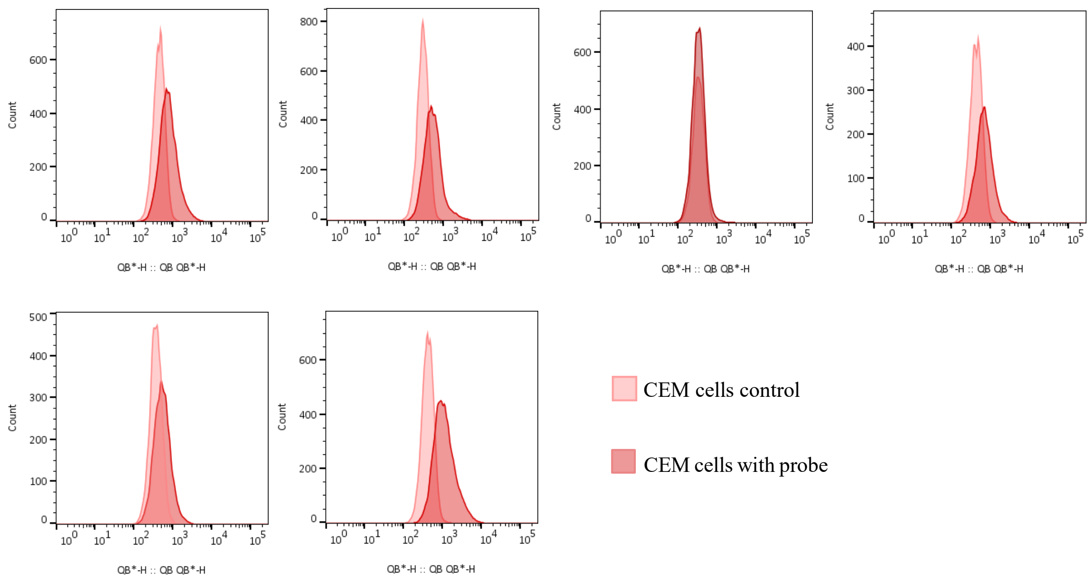

Table S10: Fluorescence enhancement after delivery of FIT probes in CCRF and Jurkat cells analyzed by flow cytometry

Probe	CCRF CEM cells					Jurkat cells				
	MFI_0	MFI	ΔMFI	SD	% increase	MFI_0	MFI	ΔMFI	SD	% increase
JK1	392	501	110	41	**28**	520	854	334	106	**64**
JK2	382	640	258	137	**68**	545	1509	964	235	**177**
JK3	382	570	188	100	**49**	545	1133	588	209	**108**
JK4	368	457	89	62	**24**	520	807	287	131	**55**
C6	440	696	256	83	**58**	704	879	175	64	**25**
C14	439	963	525	322	**120**	695	1162	467	402	**67**
C15	435	845	410	180	**94**	663	1183	521	390	**79**

MFI_0: mean fluorescence (a.u.) of control cells, MFI: mean fluorescence of cells after probe delivery, ΔMFI: mean fluorescence enhancement. SD: standard deviation of fluorescence enhancement. Data from 4-6 replicates

10. UPLC traces and MALDI-TOF-MS

C1-QB

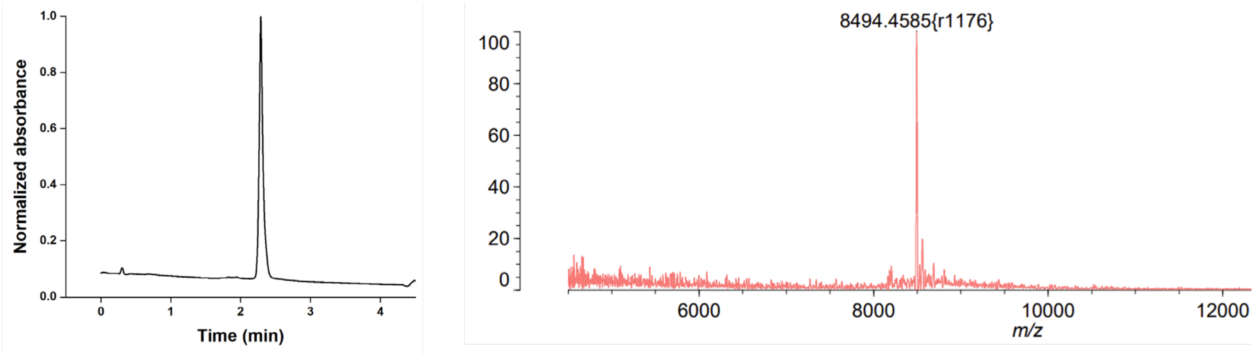

C2-QB

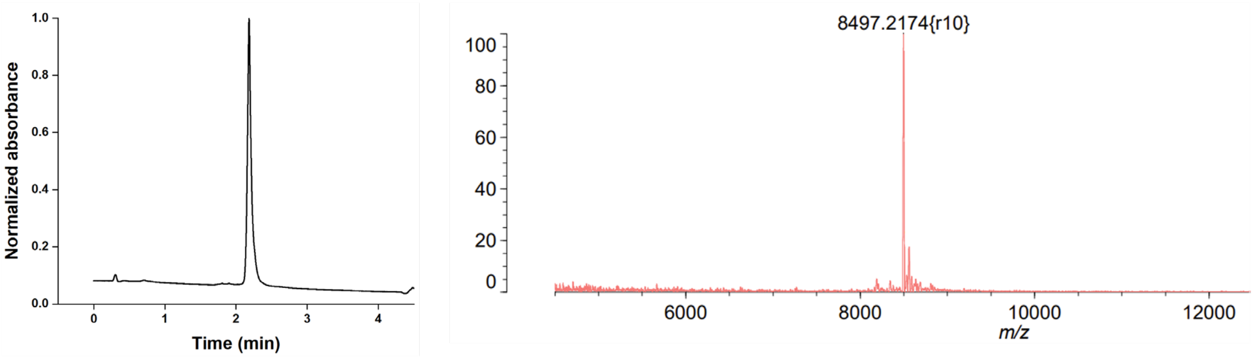

C3-QB

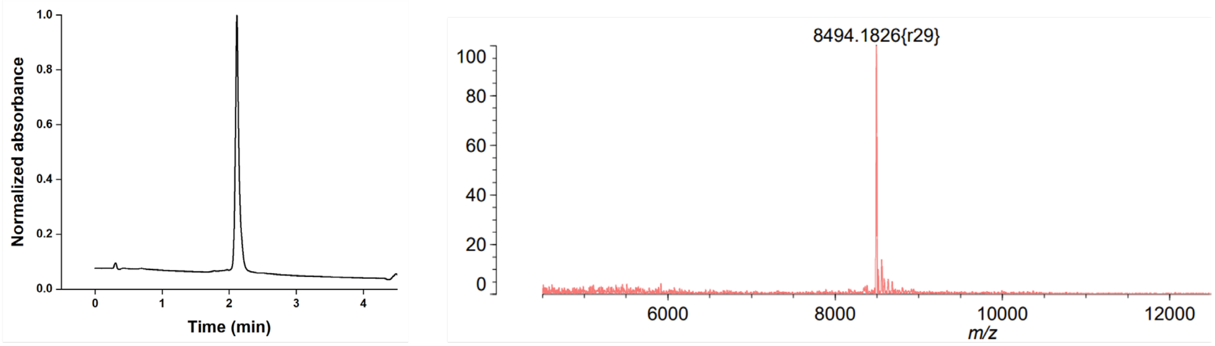

C4-QB

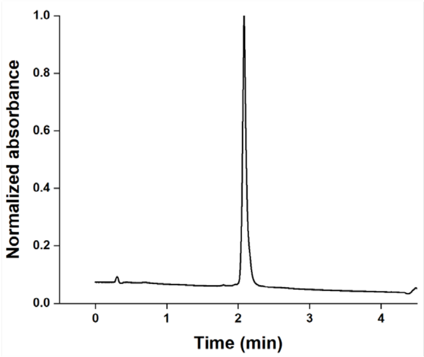

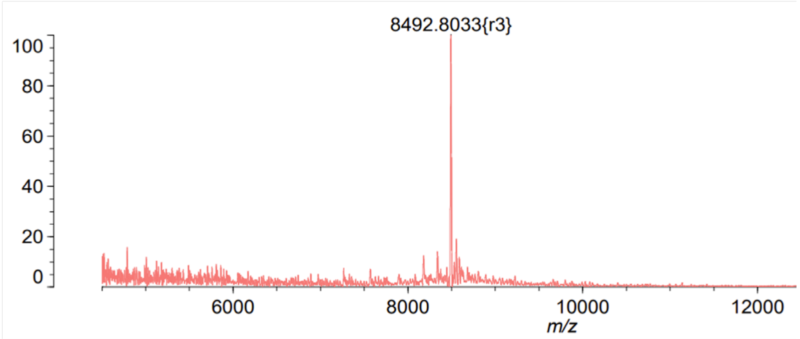

C1-AF568

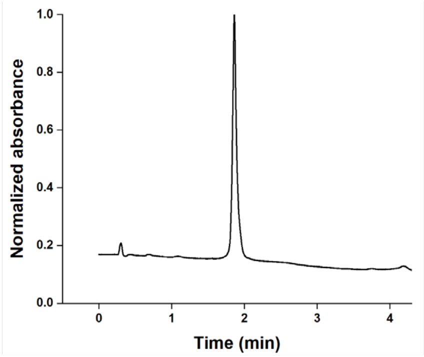

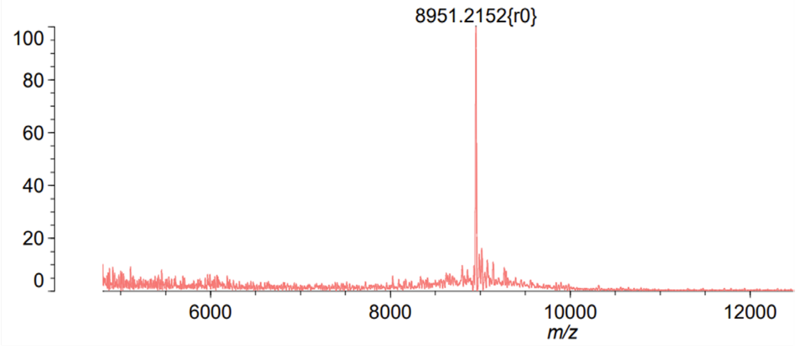

C2-AF568

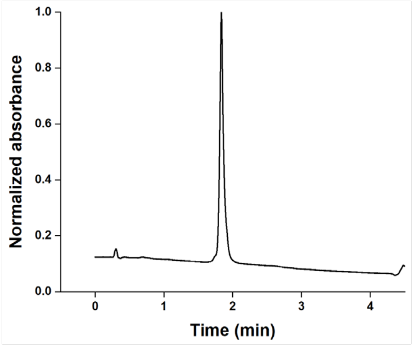

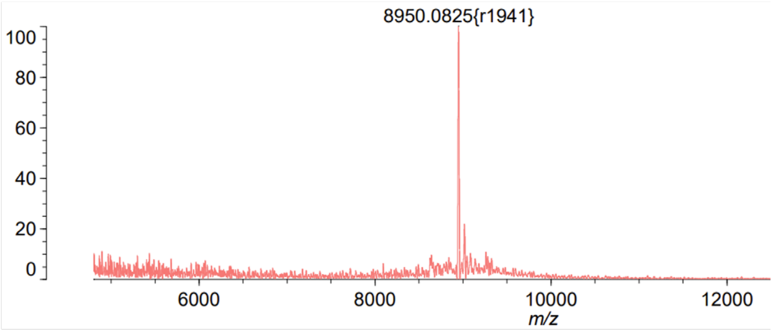

C3-AF568

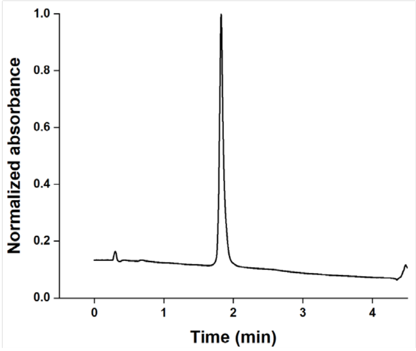

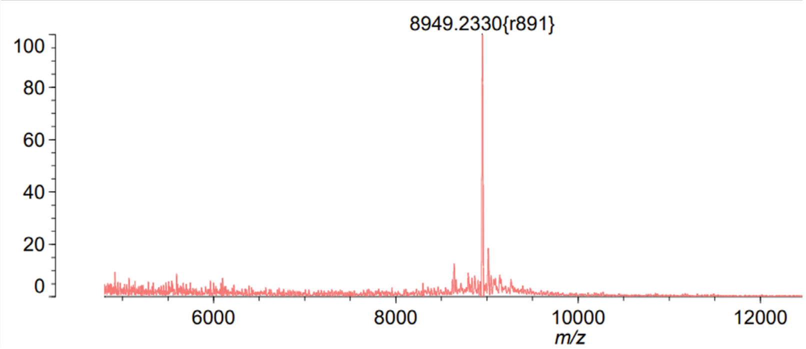

C4-AF568

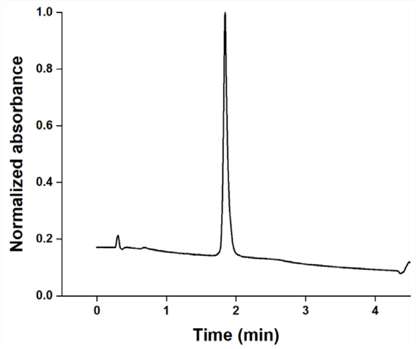

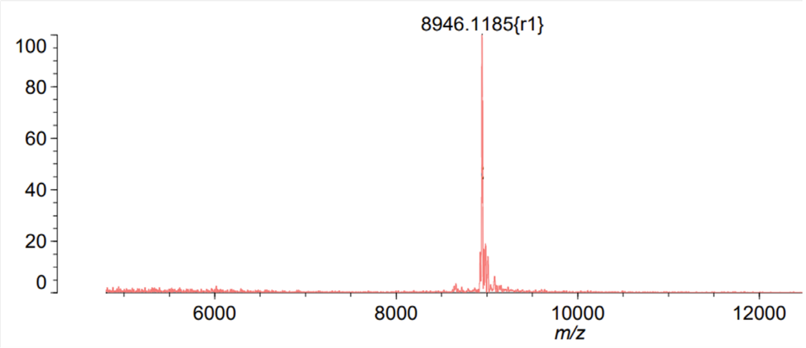

C1-AF568-QB

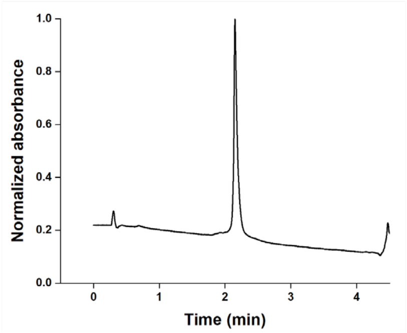

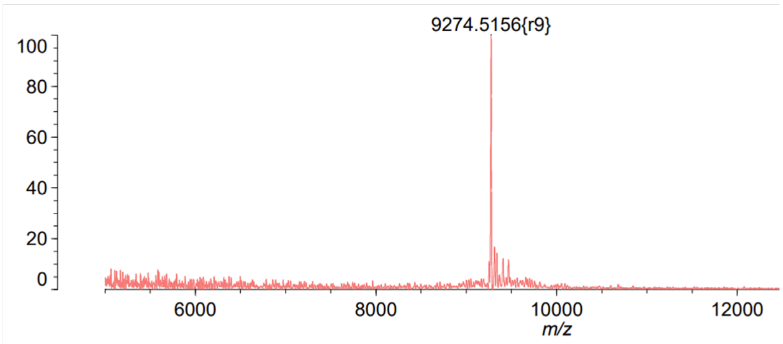

C2-AF568-QB

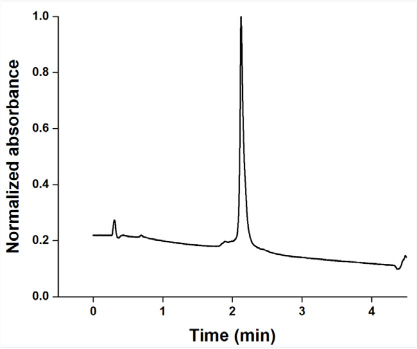

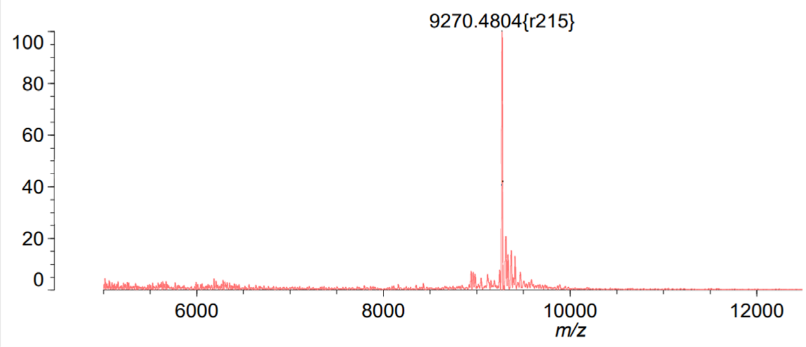

C3-AF568-QB

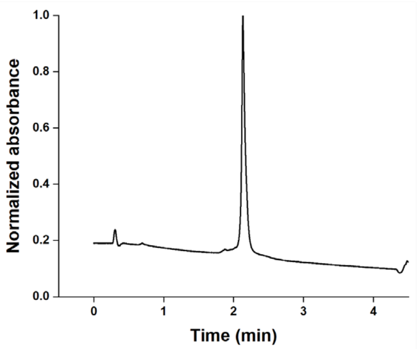

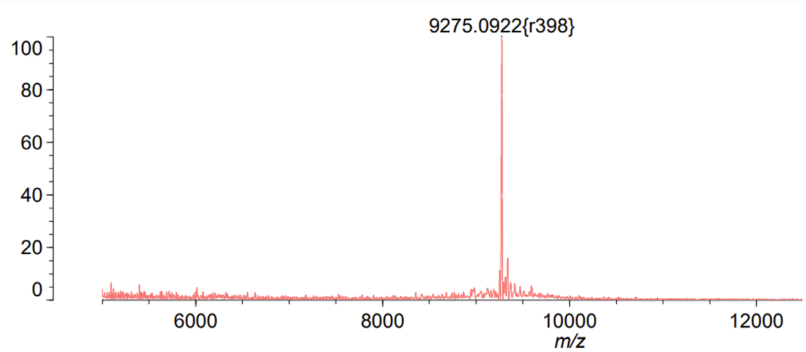

C4-AF568- QB

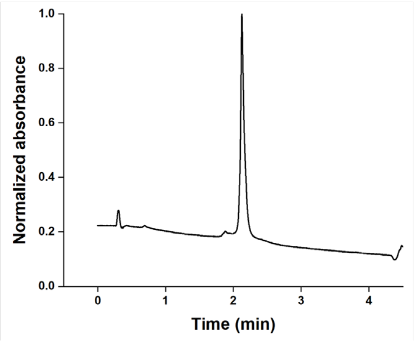

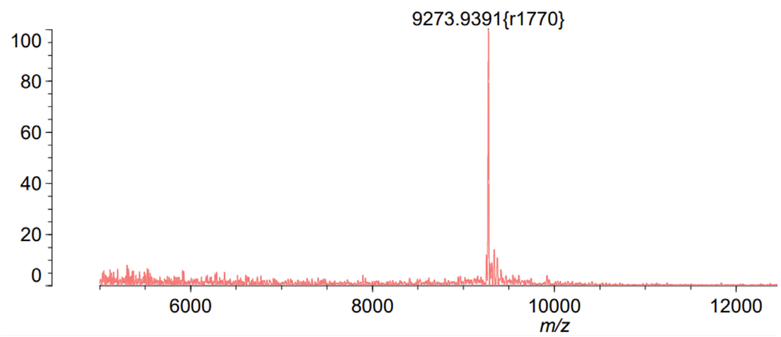

C5-2AF568

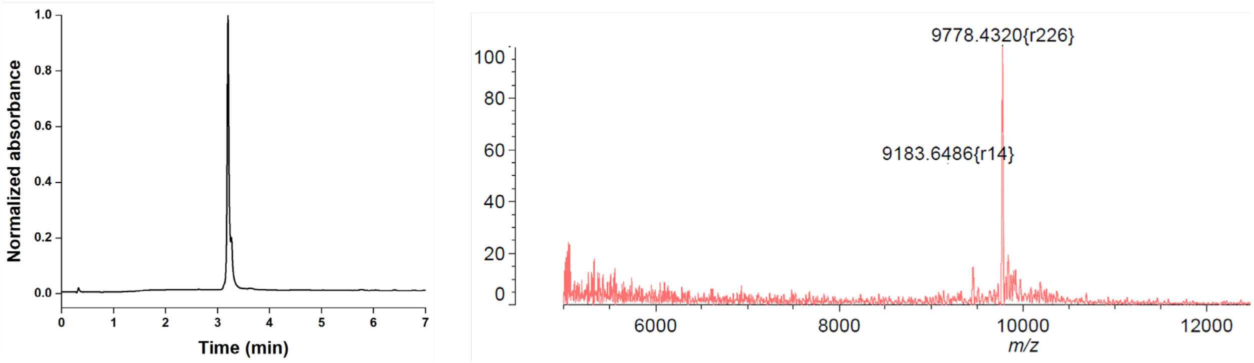

C5-2AF568-QB

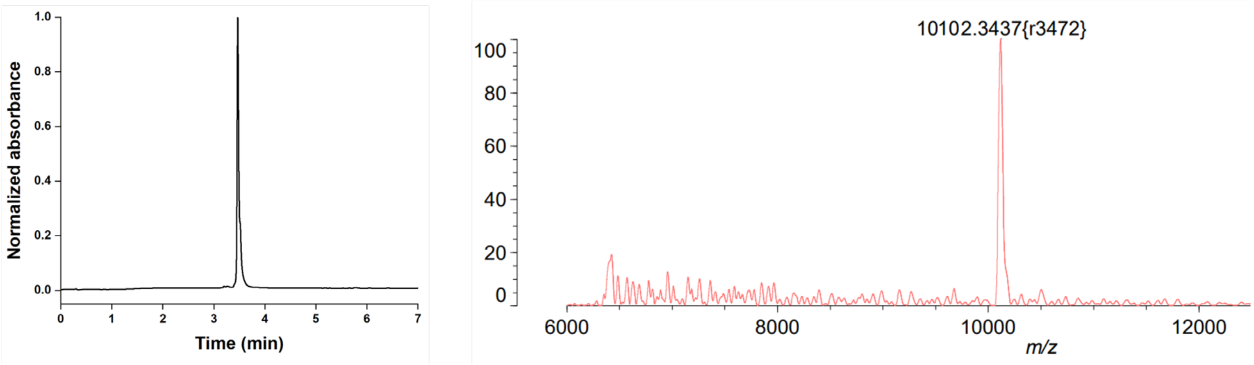

C6-2AF568

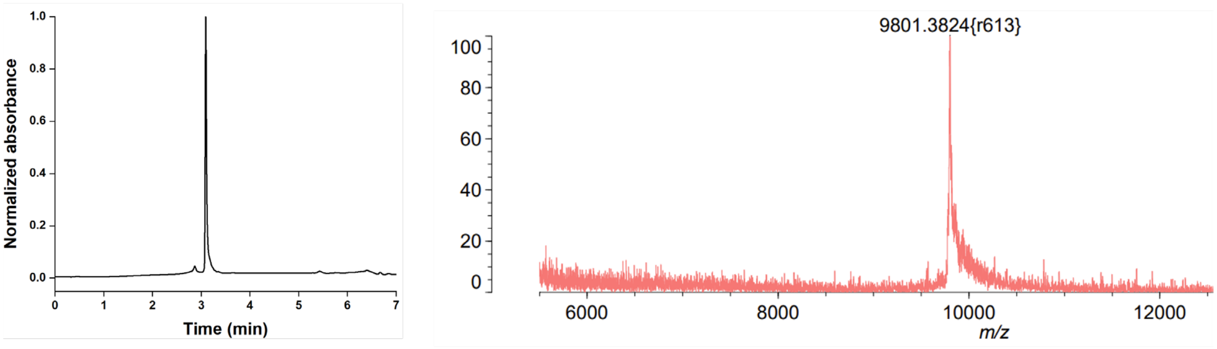

C6-2AF568-QB

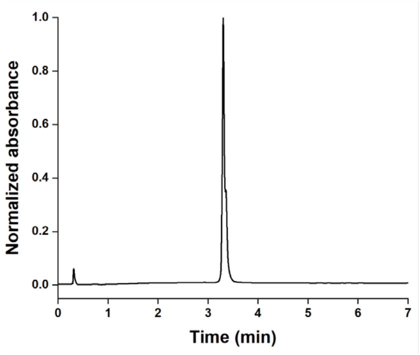

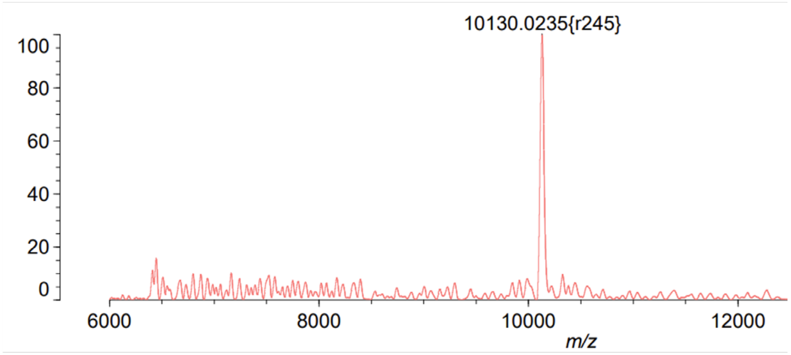

C7-2AF568

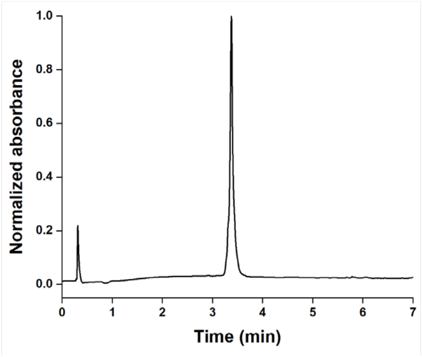

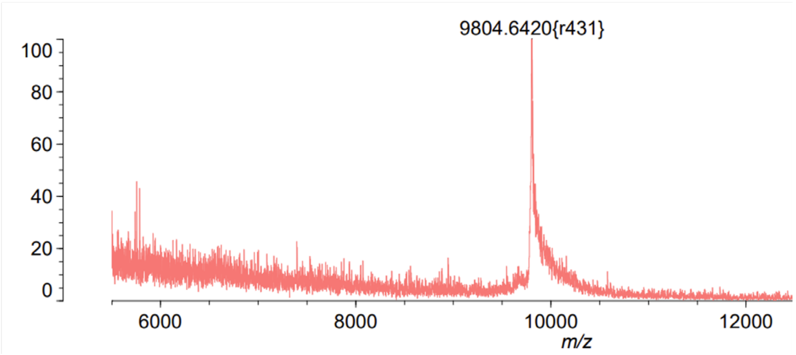

C7-2AF568-QB

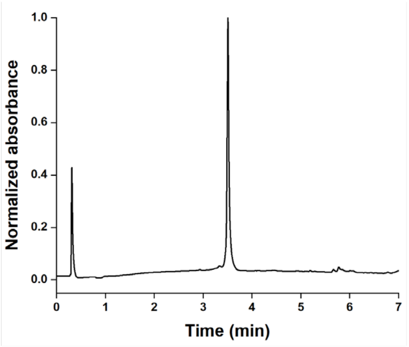

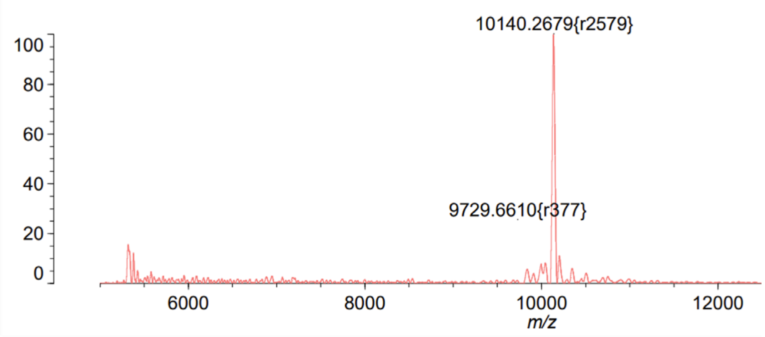

J1-2AF568

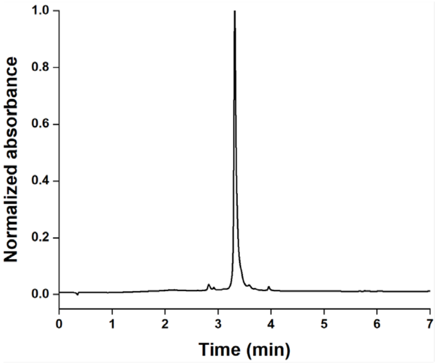

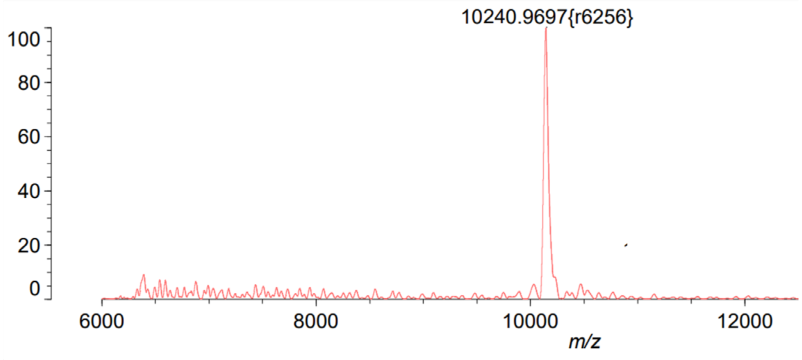

J1-2AF568-QB

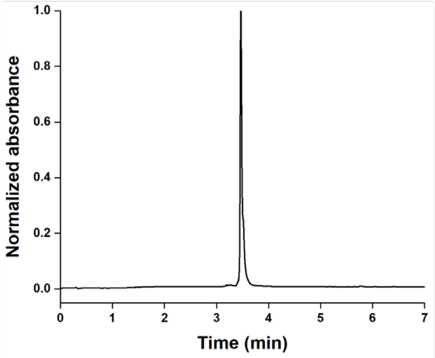

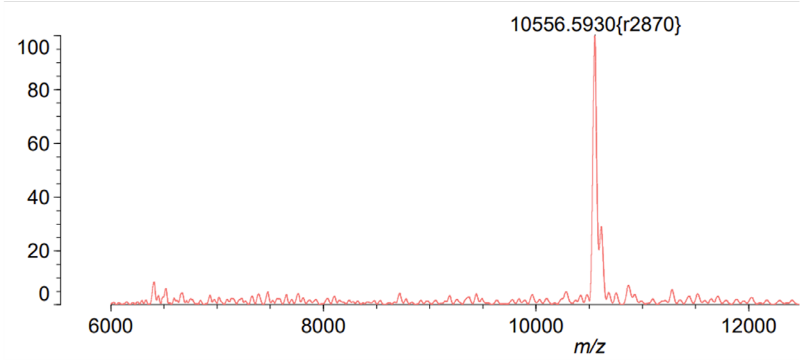

C7-QB-OMe

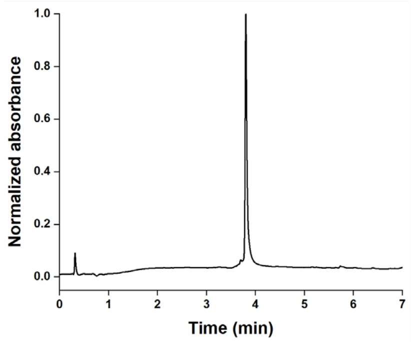

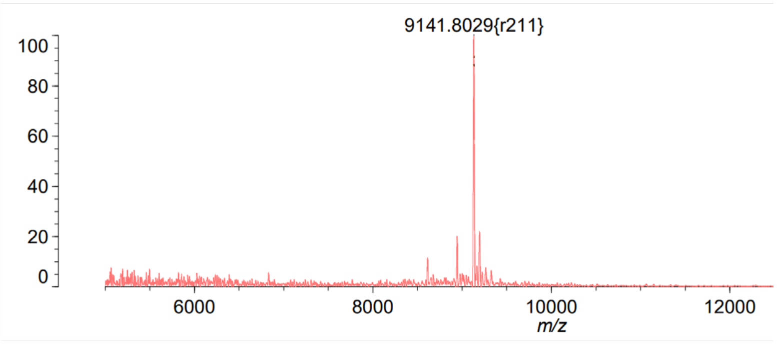

C1-AF568-QB-OMe

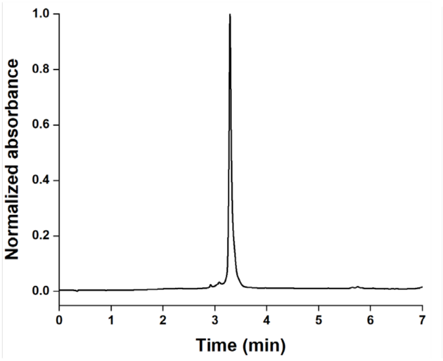

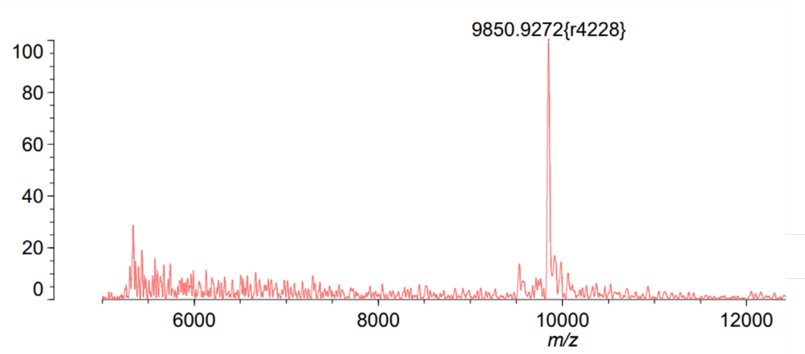

C6-2AF568-QB-OMe

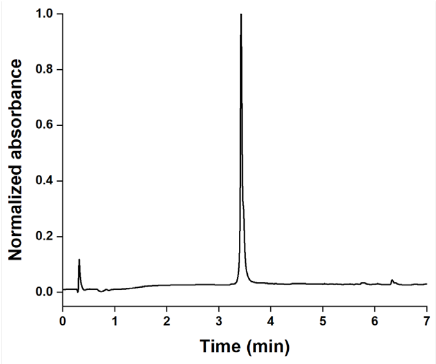

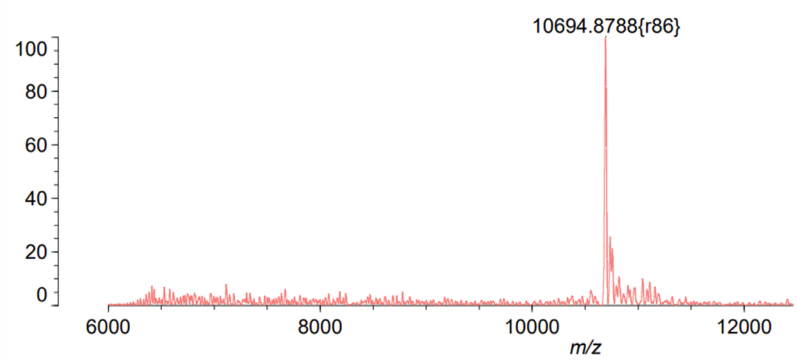

C7-2AF568-QB-OMe

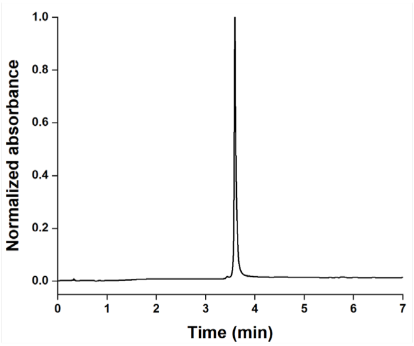

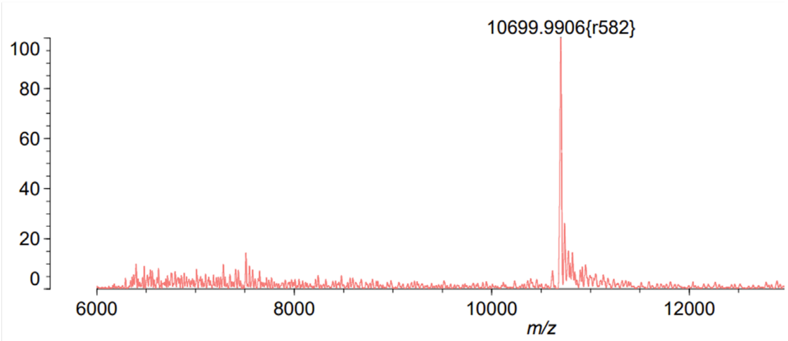

C8-2AF568-QB-OMe

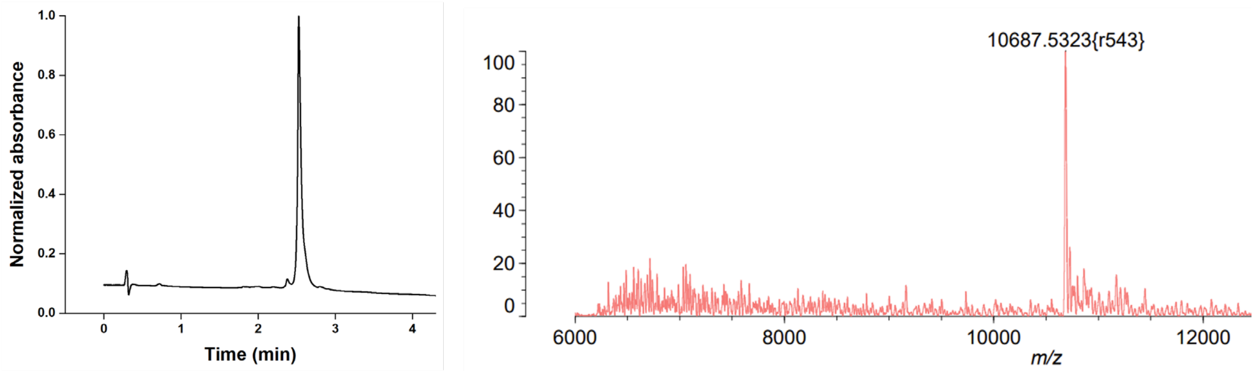

C9-2AF568-QB-OMe

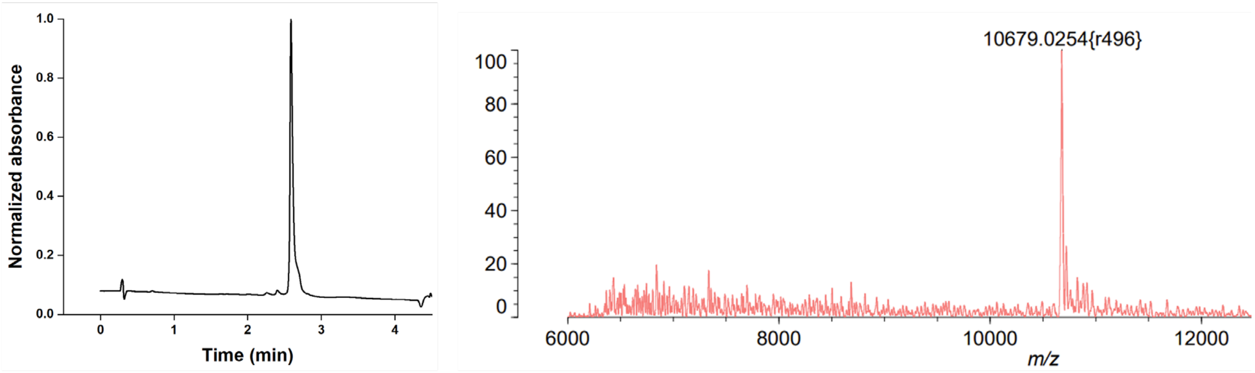

C10-2AF568-QB-OMe

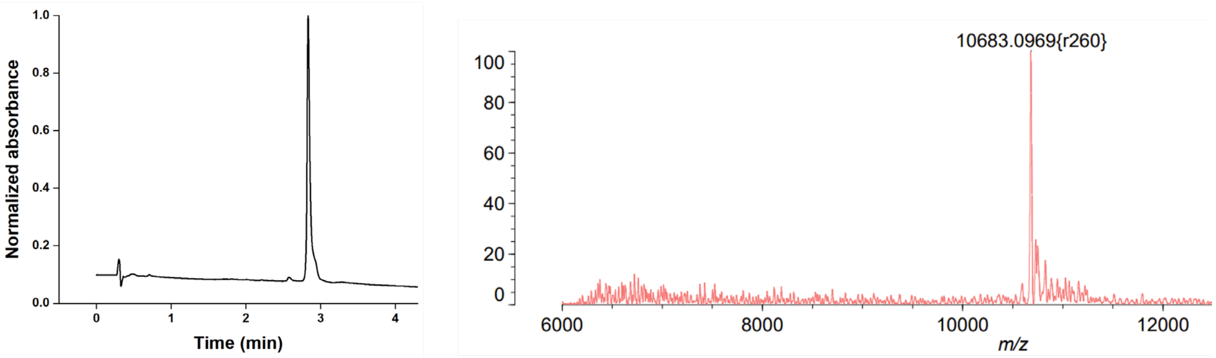

C11-2AF568-QB-OMe

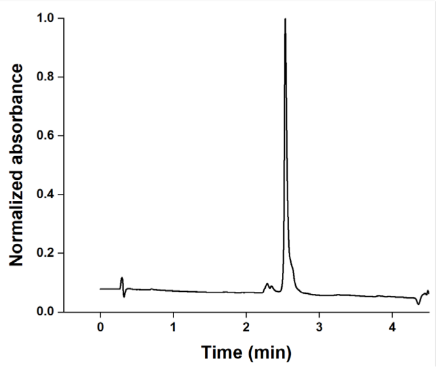

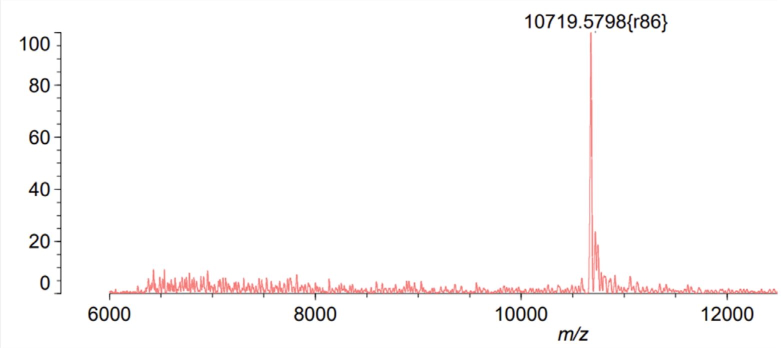

C12-2AF568-QB-OMe

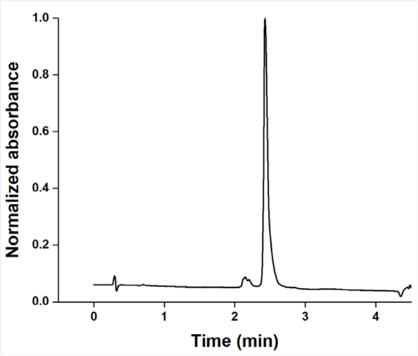

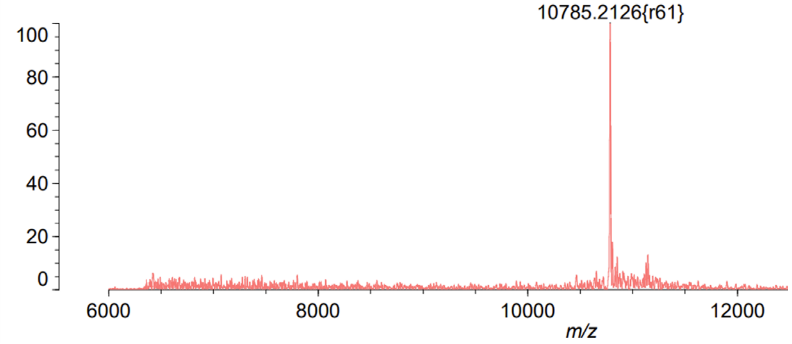

C13-2AF568-QB-OMe

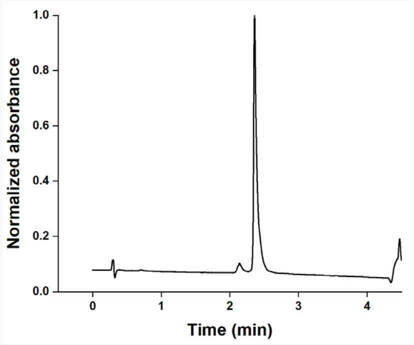

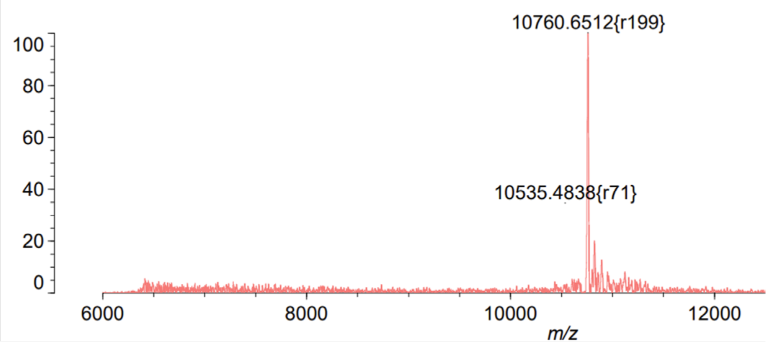

C14-2AF568-QB-OMe

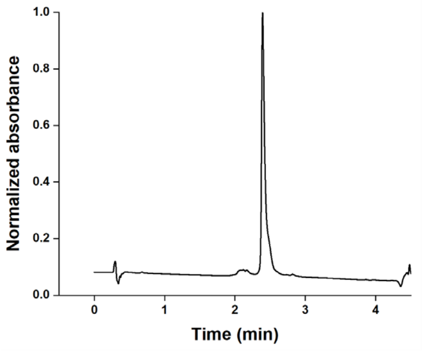

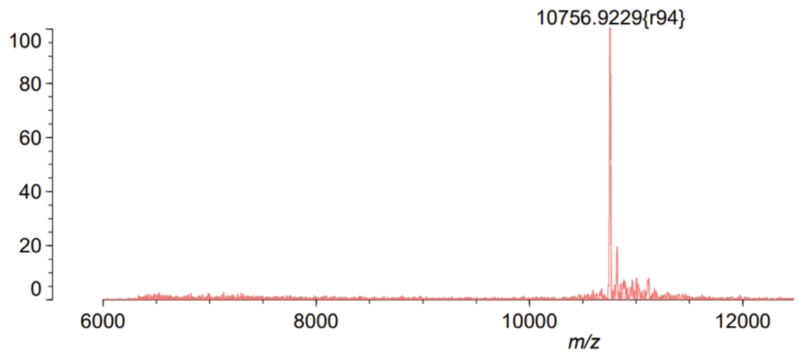

C15-2AF568-QB-OMe

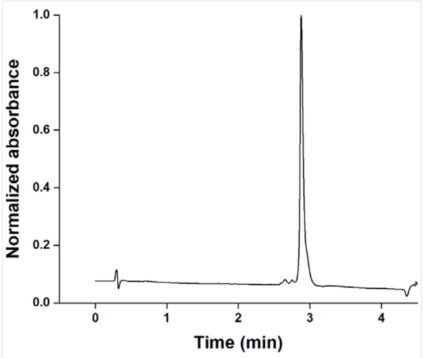

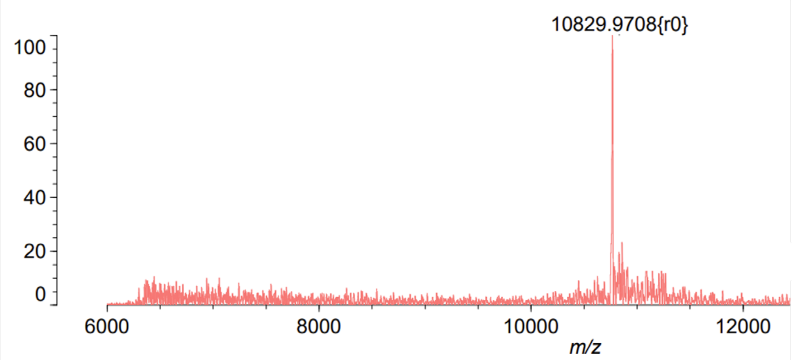

J1-2AF568-QB-OMe

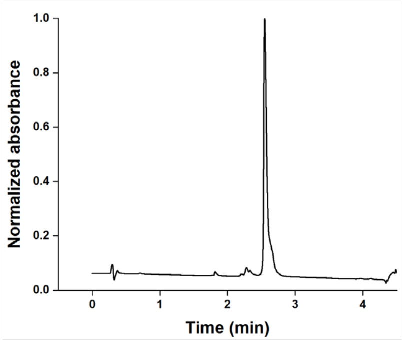

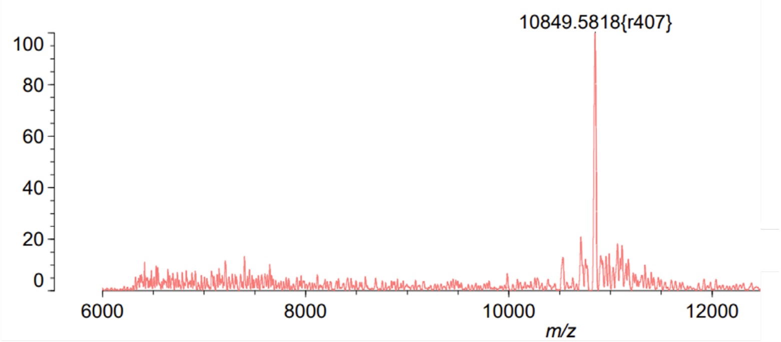

J2-2AF568-QB-OMe

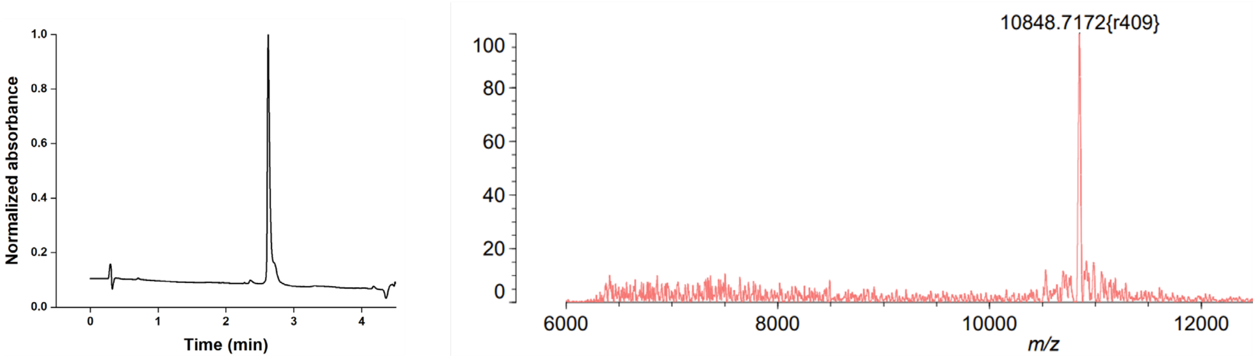

J3-2AF568-QB-OMe

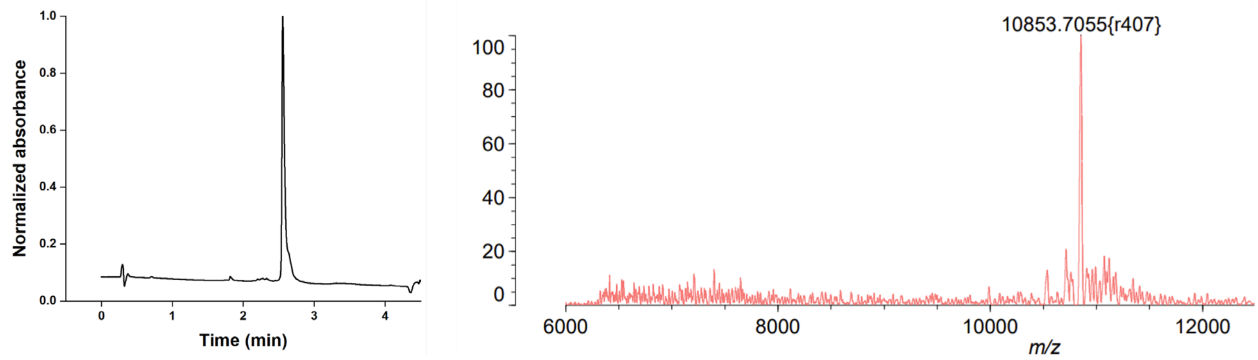

J4-2AF568-QB-OMe

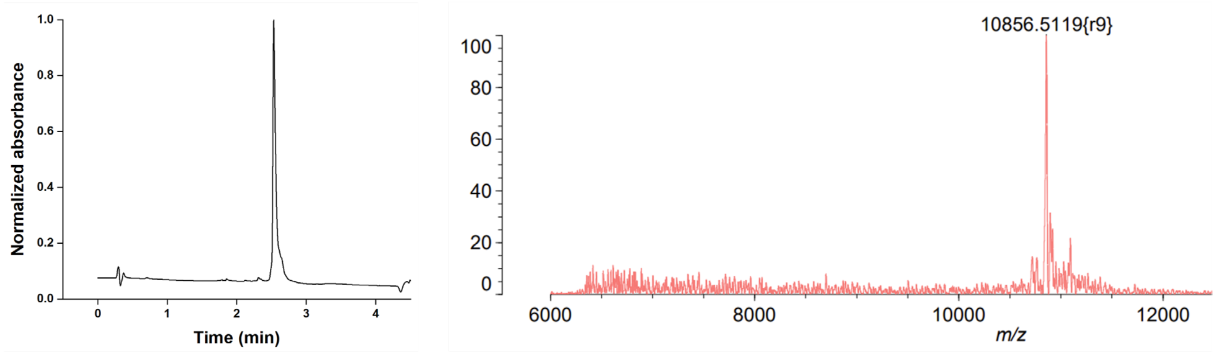

C1-AF488-TO

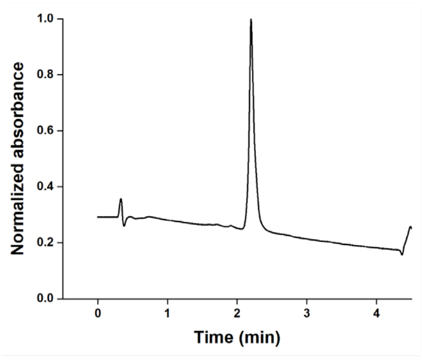

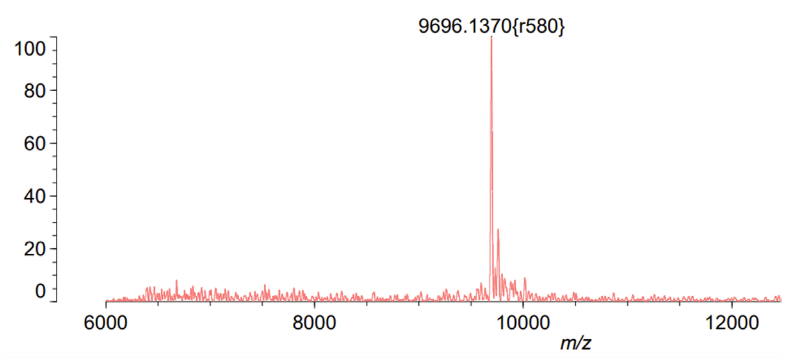

C1-AF488-TO$_{tric}$

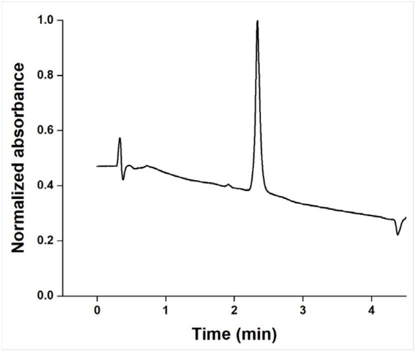

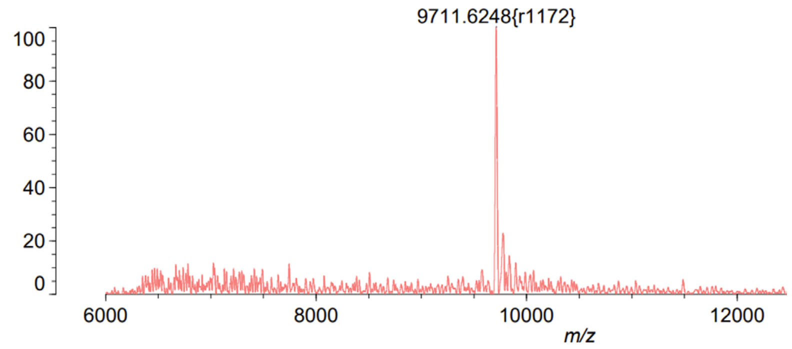

C6-2AF488-TO

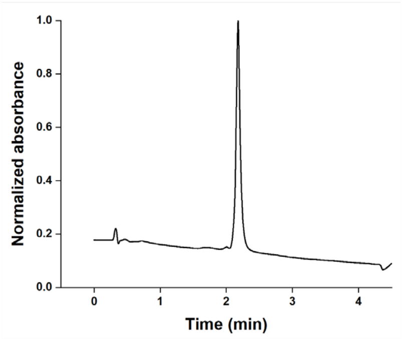

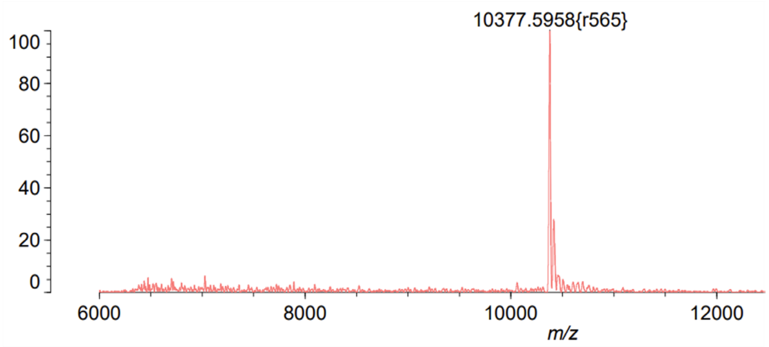

C6-2AF488-TO$_{\text{tric}}$

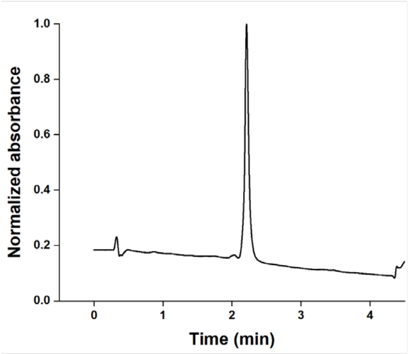

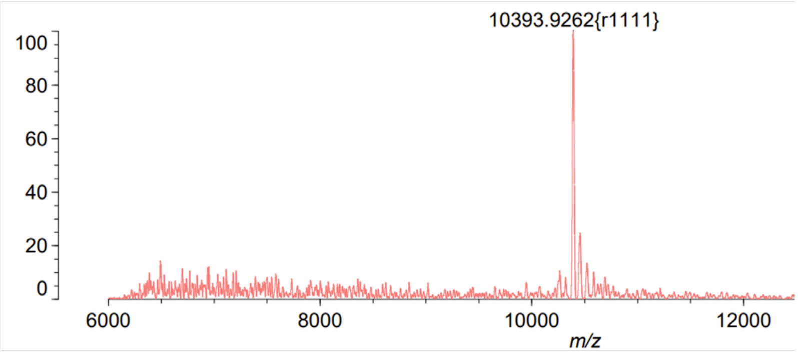

C7-2AF488-TO

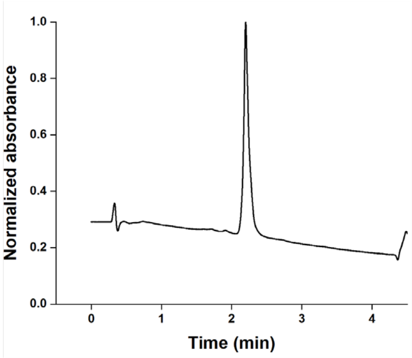

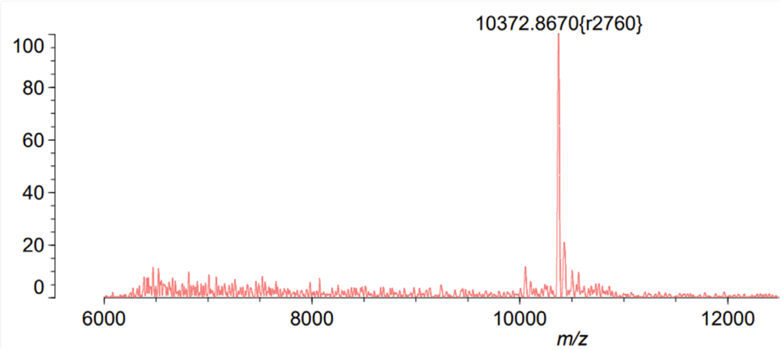

C7-2AF488-TO$_{\text{tric}}$

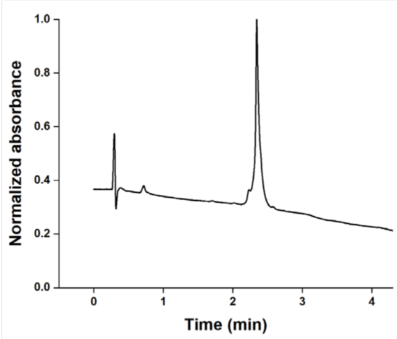

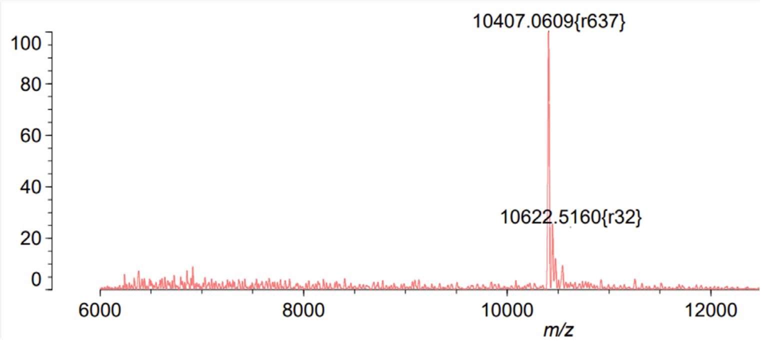

J1-2AF488-TO

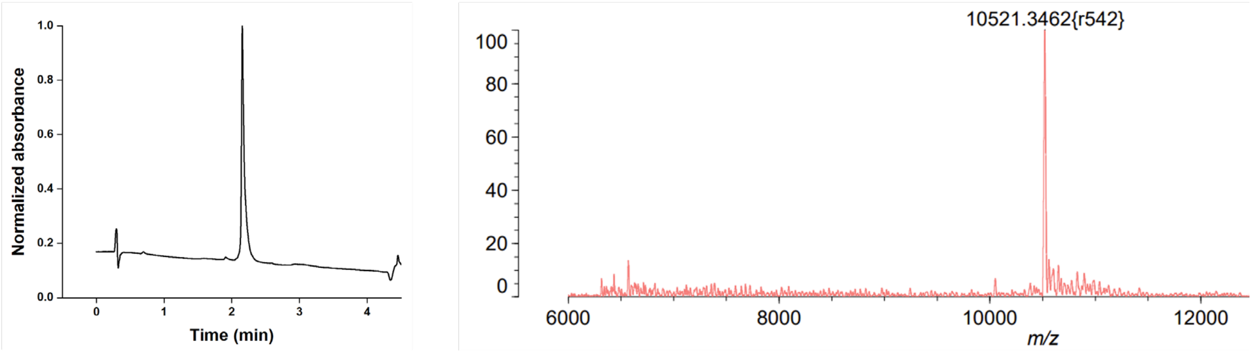

J1-2AF488-TO$_{tric}$

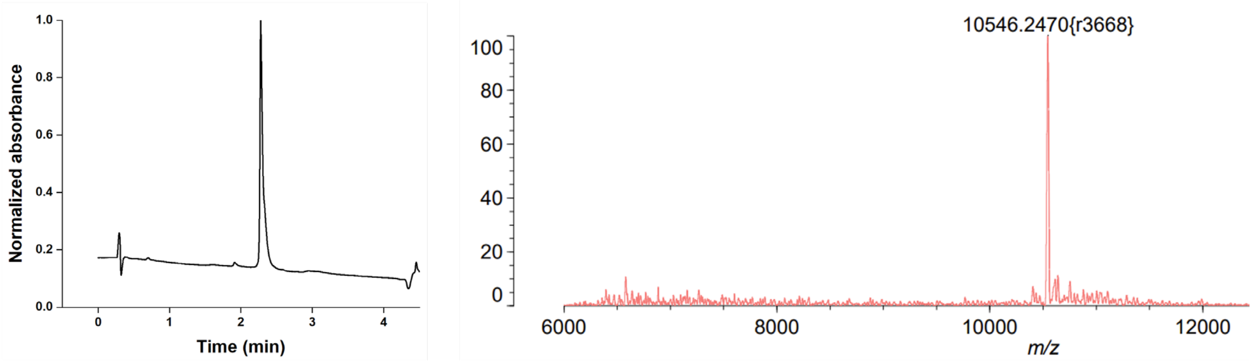

11. References

[30] R. Owczarzy, A. V. Tataurov, Y. Wu, J. A. Manthey, K. A. McQuisten, H. G. Almabrazi, K. F. Pedersen, Y. Lin, J. Garretson, N. O. McEntaggart, C. A. Sailor, R. B. Dawson, A. S. Peek, *Nucleic Acids Res.* **2008**, *36*, W163-169.

[31] F. Abendroth, A.Bujotzek, M. Shan, R. Haag, M.Weber, O.Seitz, *Angew. Chem. Int. Ed.* **2011**,50,8592 –8596

[32] "ATTO-Reagents for Click Chemistry" can be found under https://www.attotec.com/images/ATTO/Procedures/Azide_Alkyne.pdf

[33] Y. Yoshikai, D. Anatoniou, S. P. Clark, Y. Yanagi, R. Sangster, P. Van den Elsen, C. Terhorst, T. W. Mak, *Nature* **1984**, *312*, 521-524.
